# TTNPB Promotes Human Pluripotent Stem Cell‐to‐Neural Stem Cell Transition via Modulation of Chromatin Accessibility and the S‐(5′‐adenosyl)‐L‐homocysteine/Choline Metabolic Network

**DOI:** 10.1002/advs.202515648

**Published:** 2026-02-10

**Authors:** Ruilin Du, Yudi Ren, Qiaoqiao Meng, Peng Wei, Ruyu Zhu, Junjie Bao, Ye Yang, Shuo Yan, Chaorong Yue, Xueying Zhu, Shuo Cao, Chunxia Hao, Wei Sun, Yongli Song, Xihe Li, Zhimin Wu, Siqin Bao, Yanglin Chen

**Affiliations:** ^1^ Research Center for Animal Genetic Resources of Mongolia Plateau College of Life Sciences Inner Mongolia University Hohhot China; ^2^ The State Key Laboratory of Reproductive Regulation and Breeding of Grassland Livestock College of Life Sciences Inner Mongolia University Hohhot China; ^3^ National Center of Technology Innovation for Dairy Hohhot China; ^4^ Inner Mongolia Saikexing Institute of Breeding and Reproductive Biotechnology in Domestic Animal Hohhot China

**Keywords:** chromatin accessibility, metabolic reprogramming, neural stem cells, pluripotent stem cells, TTNPB

## Abstract

Efficient derivation of neural stem cells (NSCs) from human pluripotent stem cells (PSCs) is crucial in regenerative medicine. Here, we report that the combined application of the retinoic acid receptor agonist TTNPB and the GSK3β inhibitor CHIR99021 in a chemically defined medium enabled the induction of a highly advanced NSCs (ANSCs) population from PSCs. ANSCs display robust neuroectodermal gene expression and a heightened capacity for neural lineage commitment. The combination of TTNPB and CHIR99021 markedly enhanced global chromatin accessibility, particularly at neuroectoderm‐specific regulatory elements such as *PAX6* and *SOX1*, in parallel with reduced accessibility at the loci of pluripotency factors. Notably, TTNPB alone also exerts a marked effect in enhancing chromatin accessibility. Untargeted metabolomic analysis identified a distinct neural‐ectoderm associated metabolic state in ANSCs, prominently characterized by elevated choline, alongside S‐(5′‐adenosyl)‐L‐homocysteine, adenosine 5′‐diphosphate, and glutathione. Exogenous addition of these metabolites was sufficient to induce neuroectodermal marker expression, highlighting the instructive role of the metabolic network in neural fate induction. Moreover, functional studies showed that ANSCs enabled engraftment into depressed rat hippocampi and restored depression‐like behavioral deficits. Our study presents a novel small‐molecule strategy that leverages TTNPB‐centered epigenetic remodeling and metabolic reprogramming as dual mechanisms driving neural differentiation.

## Graphical abstract

A retinoic acid receptor agonist, TTNPB, drives the efficient generation of advanced neural stem cells (ANSCs) from human pluripotent stem cells. TTNPB‐centered chromatin remodeling and metabolic reprogramming, promote neuroectoderm commitment. The resulting cells show robust neural potential and functional efficacy in a rat depression model.

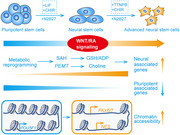

## 1 Introduction

Neural stem cells (NSCs) are undifferentiated cells of the central nervous system (CNS) with strong self‐renewal and multipotent differentiation capacities [1, 2]. These cells generate progenitors that develop into neurons, astrocytes, and oligodendrocytes [3, 4]. Present during development and in adult niches such as the hippocampus and subventricular zone [3, 5, 6, 7], NSCs support neural plasticity, repair, and regeneration, making them key models for studying brain development, disease, and potential cell therapies [8, 9]. Pluripotent stem cells (PSCs), including embryonic stem cells (ESCs) and induced pluripotent stem cells (iPSCs), are key models for studying development and can differentiate into all germ layers, offering therapeutic potential [10, 11, 12]. The transition from PSCs to NSCs reflects early neural commitment, which is regulated by signaling and transcriptional programs and has led to diverse neural induction protocols using cytokines and small molecules.

TTNPB is a potent retinoic acid receptor (RAR) agonist widely used in studies of cellular differentiation and embryonic development [10]. By activating RARα, RARβ, and RARγ, TTNPB promotes neuronal differentiation of embryonic and neural stem cells [13]. As a highly active analog of retinoic acid (RA), a vitamin A‐derived regulator of embryogenesis, TTNPB plays a key developmental role [14, 15]. In vitro, TTNPB enhances the proliferation and clonal expansion of human embryonic stem cells [16].

During CNS development, RA promotes stem cell differentiation into glial progenitors and GABAergic neurons [17, 18], while the Wnt signaling pathway regulates neurogenesis and the proliferation and differentiation of neural progenitor cells [19, 20]. In vitro, activation of Wnt signaling enhances stem cell self‐renewal and promotes the differentiation of GABAergic neurons and glial cells [21]. As key morphogens in the ventral and dorsal neural tubes, respectively, RA and Wnt signaling coordinate neural patterning and cell fate determination [22, 23, 24], suggesting that their interaction regulates NSCs proliferation and differentiation during neural development. Efficient and scalable differentiation of human PSCs into neural stem/precursor cells has been widely achieved using RA in combination with serum‐free medium supplemented with defined growth factors, neurotrophic factors, and small molecules [25, 26, 27, 28, 29]. In our preliminary experiments [11], activation of Wnt signaling by CHIR99021 combined with supplementation of leukemia inhibitory factor (LIF) efficiently induced human PSCs to differentiate into NSCs expressing canonical markers. Given the critical role of RA signaling in neural specification [30], we speculate that TTNPB may facilitate the efficient conversion of PSCs into NSCs. However, the mechanisms underlying RA‐driven neural fate determination remain unclear, particularly regarding its potential involvement in epigenetic regulation and metabolic reprogramming during neural commitment.

To explore these possibilities, we investigated the effect of adding the RAR agonist TTNPB to the PSC‐to‐NSC transition medium. We identified novel CHIR99021/TTNPB‐induced cells, called advanced neural stem cells (ANSCs). By analyzing their distinct transcriptomes, chromatin accessibility, and metabolite profiles, we aimed to uncover the detailed mechanisms through which RA signaling and metabolites influence neural cell induction. Our findings provide new insights into how signaling molecules, epigenetic remodeling, and metabolic reprogramming collectively influence cell‐fate determination.

## 2 Results

### 2.1 Synergistic Application of TTNPB and CHIR99021 Significantly Promotes the Efficient Differentiation of Human PSCs into NSCs

**FIGURE 1:**
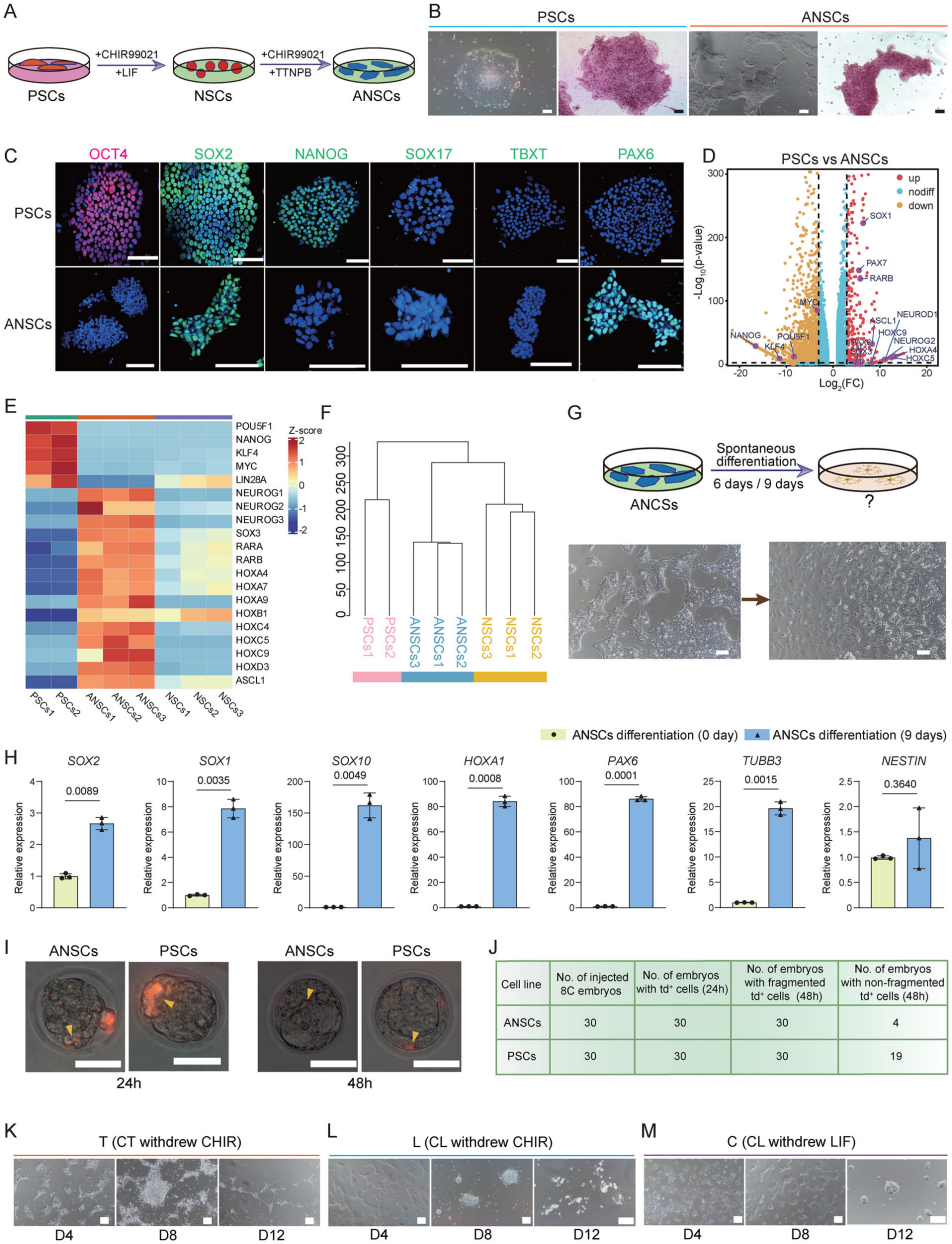
Synergistic induction of advanced neural stem cells (ANSCs) from PSCs by TTNPB and CHIR99021. (A), Schematic of the generation of advanced neural stem cells (ANSCs) from pluripotent stem cells (PSCs). (B), Morphology and alkaline phosphatase staining of PSCs (passage 20) and ANSCs (passage 50). Scale bar: 100 µm. (C), Immunofluorescence staining of pluripotency markers (*OCT4, SOX2, NANOG*) and lineage markers (*SOX17, TBXT, PAX6*) of PSCs and ANSCs. Scale bar: 50 µm. (D), Volcano plot showing differentially expressed genes (DEGs) between ANSCs and PSCs (|log_2_FC| > 1, *P* < 0.05). (E), Heatmap illustrating the expression levels of pluripotency genes, neuroectodermal genes, genes associated with retinoic acid (RA) signaling, and downstream target genes of the RA signaling pathway in ANSCs and PSCs. (F), Hierarchical clustering of transcriptomic profiles from PSCs, NSCs, and ANSCs (distance metric: 1‐ Spearman correlation coefficient). (G), Schematic and morphology on day 9 of ANSCs during spontaneous differentiation in N2B27 medium. Scale bar: 100 µm. (H), RT‐qPCR analysis of *SOX2, SOX1, SOX10, HOXA1, PAX6, TUBB3*, and *NESTIN* expression in ANSCs before (Day 0) and after spontaneous differentiation (Day 9). Data were normalized to *GAPDH*. Error bars represent mean ± SD. (n = 3 biological replicates). *P* values were determined using two‐tailed Student's *t*‐tests. (I), Representative images of mouse 8‐cell embryos at 24 and 48 h post‐injection with ANSCs or PSCs. Yellow arrows indicate the injected cells carrying the tdTomato fluorescent protein. Scale bar: 100 µm. (J), Cell counts of ANSCs and PSCs contributing to mouse embryos were performed separately. (K), Representative images of ANSCs treated with TTNPB alone (CHIR99021 withdrawal). T: TTNPB; CHIR: CHIR99021. Scale bars: 100 µm. (L), Representative images of NSCs treated with LIF alone (CHIR99021 withdrawal). L: LIF (leukemia inhibitory factor). Scale bars: 100 µm. (M), Representative images of NSCs treated with CHIR99021 alone (LIF withdrawal). C: CHIR99021. Scale bars: 100 µm.

We had previously observed that the Wnt activator CHIR99021 and LIF, in a chemically defined medium (N2B27), could differentiate NSCs from PSCs [11]. To optimize the induction conditions, we replaced LIF with TTNPB in the N2B27 medium, aiming to further enhance NSCs induction (Figure 1A). As the effective concentration of CHIR99021 had been established in previous studies, we adopted the previously reported dose of 3 µm. To investigate whether different concentrations of TTNPB would lead to varying degrees of ectodermal gene activation, we used TTNPB at concentrations of 0.5, 1, 2, and 10 µm (Figure S1A). Despite previous reports indicating that high concentrations of TTNPB possess cytotoxicity [31], no significant cell death or morphological differences were observed across the tested concentration range. RT‐qPCR analysis revealed that even at 0.5 µm, TTNPB was sufficient to significantly upregulate ectodermal genes such as *SOX2*, *PAX6*, and *SOX1* compared with PSCs (Figure S1B). Therefore, this concentration was selected for further use in our study, and the TTNPB/CHIR99021‐induced cells were designated ANSCs.

ANSCs exhibited long‐term self‐renewal capacity (passage > 50), as demonstrated by consistent alkaline phosphatase (AP) staining positivity and stable karyotype analysis (Figure 1B; Figure S1C). Upon treatment with TTNPB and CHIR99021, colony morphology shifted from large and rounded to irregular in both NSCs and PSCs (Figure 1B). ANSCs showed significantly lower expression of the pluripotency genes *OCT4* and *NANOG* than PSCs, which expressed a full panel of pluripotency markers (Figure S1B). Conversely, the expression of the neuroectoderm‐associated genes *SOX2* and *PAX6*, key regulators of neural cell differentiation, was significantly elevated in ANSCs compared to both PSCs and NSCs (Figure 1C; Figure S1D).

To confirm our findings, we validated the establishment process using another human embryonic stem cell line, H9, and an induced pluripotent stem cell line, Z1. To distinguish NSCs and ANSCs derived from different cell lines, unless otherwise specified, the PSCs, ANSCs, and NSCs used in this study were all derived from the human embryonic stem cell line W24.

Our results showed that ANSCs and NSCs derived from both H9 and Z1 displayed outcomes fully consistent with those described above, including positive AP staining (Figure S2A,B), downregulation of pluripotency genes and proteins (OCT4 and NANOG), and upregulation of ectodermal genes and proteins (SOX2 and PAX6) (Figure S2C–F).

To explore the molecular characteristics of ANSCs, we performed RNA sequencing analysis. The results revealed that, compared with PSCs and NSCs, multiple genes associated with neuroectodermal identity, including *PAX7*, *SOX1*, and members of the NEUROG family, were significantly upregulated in ANSCs (Figure 1D,E). In addition, genes involved in RA signaling, such as *RARB* and its downstream targets *HOXA4* and *HOXC9*, were also elevated. Conversely, key pluripotency markers, including *NANOG*, *POU5F1*, *KLF4, MYC*, and *LIN28A* were markedly downregulated in ANSCs (Figure 1D,E). Hierarchical clustering further demonstrated that ANSCs and NSCs formed a distinct cluster, indicating similar transcriptomic features, and were markedly divergent from PSCs (Figure 1F). Consistent with these observations, similar results were obtained in ANSCs derived from H9 cells (H9‐ANSCs). H9‐ANSCs, comparable to ANSCs (derived from the W24 cell line), exhibited a transcriptional profile characterized by high expression of neuroectoderm‐associated genes (Figure S2G–I). Genes upregulated in H9‐ANSCs were also significantly enriched in pathways related to axon development, axon guidance, and hedgehog signaling (Figure S2J–L). Hierarchical clustering analysis further indicated that H9‐ANSCs clustered together with H9‐NSCs and that both cell types shared 6808 differentially expressed genes relative to uninduced H9 cells (Figure S2M,N).

### 2.2 Loss of Pluripotency and Acquisition of Neural Fate Bias in ANSCs

To further investigate the differentiation potential of ANSCs, we allowed spontaneous ANSCs differentiation for six and nine days. After differentiation, ANSCs displayed a morphology resembling that of neuroectodermal cells, and some cells exhibited extended neurite‐like projections (Figure 1G). On day 6 of differentiation, *SOX2*, *SOX1*, *SOX10*, *HOXA1*, and *PAX6* were significantly upregulated in ANSCs (Figure S3A). By day 9, compared to earlier time points, a broader set of ectodermal and neural lineage markers, including *SOX2*, *SOX1*, *SOX10*, *HOXA1*, *PAX6*, *TUBB3*, and *NESTIN*, were significantly upregulated (Figure 1H). Immunofluorescence staining confirmed the strong propensity of ANSCs to spontaneously differentiate into neural cells (Figure S3B). To further compare the differentiation potential between ANSCs and NSCs, we assessed the expression of ectodermal lineage genes after differentiation. Our results showed that *TUBB3*, *SOX10*, *SOX2*, *PAX6*, *NESTIN*, and *HOXA1* were consistently expressed at significantly higher levels in ANSCs than in NSCs on days 6 and 9, indicating that ANSCs possess a stronger differentiation capacity and a greater propensity toward ectodermal lineage commitment (Figure S3C,D).

In addition, NSCs and ANSCs derived from Z1 and H9 lines were also subjected to spontaneous differentiation for nine days, followed by the assessment of a broader set of ectodermal and neural lineage markers (Figure S4A). The results showed that both H9‐ and Z1‐derived ANSCs retained their capacity to differentiate into neural cells under spontaneous differentiation conditions (Figure S4B–H).

Conversely, to evaluate the developmental potential of ANSCs, we performed interspecies chimera experiments by injecting ANSCs into mouse 8‐cell stage embryos. Thirty embryos were injected with ANSCs. At both 24 and 48 h post‐injection, ANSCs showed significantly lower viability than PSCs in incompatible microenvironments; moreover, they exhibited more pronounced fragmentation compared with their PSCs counterparts under identical experimental conditions. (Figure 1I,J; Figure S4I).

### 2.3 TTNPB Synergizes with CHIR99021 to maintain NSCs Identity but Lacks Independent Inductive Capacity

Next, we explored the necessity of CHIR99021 and LIF in maintaining stem cell viability. When CHIR99021 was removed from the ANSCs culture medium, and only TTNPB was applied, massive cell death occurred, and normal proliferation could not be maintained within 12 days (Figure 1K). Similarly, when CHIR99021 was removed from the NSCs culture medium, and only LIF was applied, cells also failed to maintain normal growth and underwent extensive death (Figure 1L). Comparatively, in the NSCs culture system, when CHIR99021 was applied alone, partial cell death was still observed, but the surviving cells formed compact and spherical colonies (Figure 1M). Additionally, ANSCs treated with TTNPB alone exhibited non‐specific tropism, showing no bias toward any of the three germ layers (ectoderm, mesoderm, and endoderm). NSCs treatment with LIF alone upregulated mesodermal and endodermal gene expression (Figure S5A,B). These results indicated that CHIR99021 was indispensable for the survival and maintenance of both NSCs and ANSCs under the current culture conditions.

Furthermore, we assessed the effects of different small‐molecule/cytokine combinations on cell proliferation by performing cell counts and generating growth curves. The data revealed that neither LIF nor TTNPB alone could support normal cell proliferation. However, combined treatment with TTNPB and CHIR99021 significantly promoted cell growth (Figure S5C). In addition, OD450 measurements under varying concentrations of LIF showed that cell proliferation was most robust at 10 ng/mL (Figure S5C). Given the neural differentiation tendency of ANSCs under spontaneous differentiation conditions, the ANSCs were further differentiated into cranial placode (CP) cells using a previously reported protocol (Figure S5D) [32]. The resulting cells exhibited co‐expression of CP marker genes *SOX2*, *PAX6*, and *NESTIN*, indicating that the differentiated cells exhibited characteristics of CP cells (Figure S5E,F).

Collectively, these results demonstrated that the combination of TTNPB and CHIR99021 in a defined N2B27 medium efficiently drove the differentiation of PSCs into ANSCs. Notably, TTNPB amplified the neuroectodermal‐inducing effect of CHIR99021, leading to a more robust and lineage‐specific neural commitment with enhanced ectodermal features.

### 2.4 TTNPB Enhances Global Chromatin Accessibility and Transcriptional Activation of Neural Ectodermal Genes in ANSCs

**FIGURE 2:**
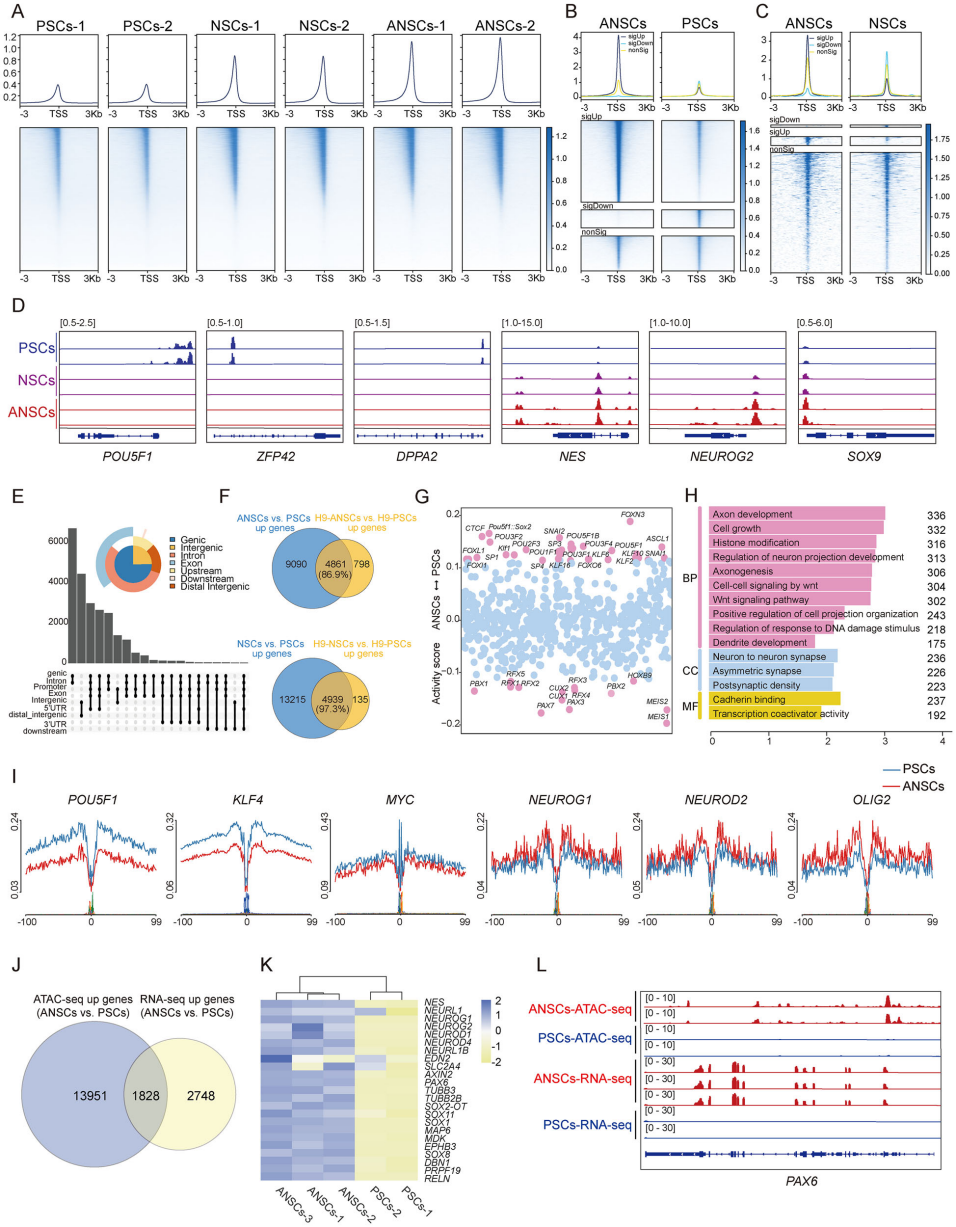
ANSCs exhibit distinct chromatin accessibility landscapes. (A), ATAC‐seq analysis of PSCs, NSCs, and ANSCs. ATAC‐seq signals at Refseq genes as normalized CPM (counts per million). Heatmaps showing the landscapes of peaks around the transcription start sites (TSSs). (B), Signal intensity distribution of differential chromatin accessibility between ANSCs and PSCs (sigUp: significantly upregulated; sigDown: significantly downregulated; nonSig: not significant). (C), Signal intensity distribution of differential chromatin accessibility between ANSCs and NSCs (categories as in B). (D), Integrative Genomics Viewer (IGV) snapshots showing ATAC‐seq signals at pluripotency‐ and neuroectodermal‐ associated gene loci. (E), Genomic distribution of differentially enriched ATAC‐seq peaks (ANSCs vs. PSCs). (F), Venn diagram illustrating the overlap of upregulated genes in ANSCs vs. PSCs and H9‐ANSCs vs. H9‐PSCs from ATAC‐seq data (upper panel). Venn diagram illustrating the overlap of upregulated genes in NSCs vs. PSCs and H9‐NSCs vs. H9‐PSCs from ATAC‐seq data (lower panel). (G), Scatter plot of chromatin accessibility activity scores for ANSCs vs. PSCs. (H), Gene Ontology (GO) enrichment analysis of genes showing increased chromatin accessibility in ANSCs compared to PSCs. BP: biological process; CC: cellular component; MF: molecular function. (I), Chromatin footprints of pluripotent and neuroectodermal transcription factors in ANSCs vs. PSCs. Red: ANSCs; Blue: PSCs. (J), Venn diagram showing the overlap between upregulated genes from ATAC‐seq and RNA‐seq (ANSCs vs. PSCs). (K), Heatmap showing neuroectodermal gene expression in ANSCs and PSCs. (L), IGV snapshots showing ATAC‐seq and RNA‐seq signals at the *PAX6* locus.

Previous studies demonstrated that TTNPB, an RA receptor agonist, enhances the effects of other small molecules during somatic cell reprogramming [33, 34]. Consistently, we found that adding TTNPB into our induction medium augmented the neural‐inductive effect of CHIR99021, promoting differentiation. To explore the mechanism underlying this synergistic effect, we performed ATAC‐seq on PSCs, NSCs, and ANSCs to evaluate their chromatin accessibility landscape. High‐quality ATAC‐seq data were obtained, characterized by clear nucleosol periodicity and strong enrichment around transcription start sites (TSS), indicating robust library complexity and sequencing quality (Figure S6A). Clustering analysis revealed distinct chromatin accessibility profiles between ANSCs and PSCs (Figure S6B). Notably, heatmaps and average signal plots showed that ANSCs exhibited the strongest signal enrichment around ± 3 kb of TSSs compared to NSCs and PSCs, suggesting globally chromatin remodeling during the differentiated process (Figure 2A).

Compared with PSCs, ANSCs showed a markedly higher number of upregulated (23 503) than downregulated (3508) peaks in the promoter regions (Figure 2B). A similar trend was observed when comparing ANSCs to NSCs, with 3758 peaks upregulated and only 363 downregulated peaks (Figure 2C). As expected, genes associated with pluripotency, including *POU5F1*, *ZFP42*, and *DPPA2*, exhibited decreased chromatin accessibility in ANSCs and NSCs, whereas genes associated with neuroectodermal development, such as *NES*, *NEUROG2*, and *SOX9* displayed significantly elevated accessibility in ANSCs (Figure 2D), suggesting extensive chromatin remodeling during neural lineage commitment.

Further analysis of the differential peak distribution revealed that regions of increased accessibility in ANSCs (vs. PSCs) were mainly located at the promoter and distal intergenic regions (Figure 2E), while in ANSCs vs. NSCs, most peaks were found in intergenic regions (Figure S6C).

ATAC‐seq analysis of H9‐PSCs, H9‐NSCs, and H9‐ANSCs revealed that H9‐ANSCs exhibited enhanced global chromatin accessibility compared to H9‐PSCs and H9‐NSCs (Figure S6D). Differentially accessible peaks were predominantly enriched in the promoter and exon regions (Figure S6E–J). Notably, among the 5659 genes corresponding to the upregulated peaks in the H9‐ANSCs vs. H9‐PSCs comparison, 86.9% were also identified in the gene set associated with the up‐regulated peaks in the ANSCs vs. PSCs (W24) comparison. Similarly, 97.3% of the 5074 genes linked to the upregulated peaks in H9‐NSCs vs. H9‐PSCs were shared with those in the NSCs vs. PSCs (W24) comparison (Figure 2F). These findings highlighted the strong consistency and robustness of our datasets.

### 2.5 Transcription Factor Landscape Reveals Neural Activation and AP‐1 Engagement in ANSCs

Transcription factor activity scoring showed that neural lineage‐associated factors, including *PAX3*, *PAX7*, and *HOXB9* were highly active in ANSCs, whereas pluripotency‐related regulators such as *FOXL1*, *POU5F1::SOX2*, and *KLF1* displayed high activity in PSCs (Figure 2G). Upon analyzing the effect of TTNPB, ANSCs exhibited increased activity of Activator Protein‐1 (AP‐1) complex components [35, 36], including *JUNB*, *JUND*, *FOS*, *FOSL1*, and *FOSL2*, compared to NSCs. This suggested that RAR/retinoid X receptor (RXR) signaling promoted neural differentiation by modulating AP‐1 transcription factor activity, which is consistent with the reported role of AP‐1 in vertebrate neurodevelopment [37]. Conversely, C2H2‐type zinc finger transcription factors, like the *KLF* family, specificity protein (Sp) family, and early growth response (EGR) family, exhibited high binding activity in NSCs, suggesting that RA signaling may not directly regulate these factors (Figure S7A). However, more evidence is required to confirm this relationship in future studies.

Moreover, Gene Ontology analysis of differentially active transcription factors (ANSCs vs. PSCs) revealed enrichment for axon development, cell growth, and histone modification (Figure 2H), whereas downregulated transcription factors (TFs) were associated with embryonic organ development and regulation of developmental processes (Figure S7B). Footprinting profiles highlighted a clear divergence in transcriptional regulatory landscapes between ANSCs and PSCs. ANSCs exhibited strong and specific footprinting enrichment at regulatory regions of neural lineage–associated genes (e.g., *NEUROG1, NEUROD2, OLIG2, SOX3, SOX1*), consistent with enhanced occupancy of neural identity–defining transcription factors. Conversely, pluripotency‐associated transcription factors, such as *POU5F1, KLF4, MYC, TBX3, TFCP2*, and *PRDM15*, showed robust footprinting signals in PSCs but were markedly reduced in ANSCs (Figure 2I; Figure S7C,D).

### 2.6 Combined RNA‐seq and ATAC‐seq Uncover Safe and Effective Chromatin Remodeling Driving Neural Fate

To comprehensively and systematically characterize the features of CHIR99021/TTNPB‐induced ANSCs, we performed an integrated analysis of RNA‐seq and ATAC‐seq data. Overlap analysis showed that 1828 genes were upregulated at the transcriptional level and exhibited increased chromatin accessibility in ANSCs vs. PSCs, including neural genes such as *PAX6*, *NES*, *NEUROD1/2/4*, and *SOX11* (Figure 2J,K). Notably, *PAX6*, a key transcription factor involved in neural ectoderm development, exhibited a dramatic increase in chromatin accessibility at its promoter region, which is consistent with its elevated expression and role in specifying ectodermal fate (Figure 2L). In addition, 21 genes were upregulated at the transcriptional level, and chromatin accessibility was increased in ANSCs compared with NSCs. Most of these genes were related to neural development (Figure S7E).

Previous studies have indicated that high chromatin accessibility can be either a cause or consequence of DNA damage or metabolic stress [38, 39, 40]. Therefore, we analyzed the expression of genes associated with DNA damage and glycolysis. Our data revealed that no genes were significantly upregulated in ANSCs, suggesting that our culture system was non‐toxic (Figure S7F,G). Together, our data revealed that the application of TTNPB enhanced CHIR99021‐mediated neural induction by globally remodeling chromatin accessibility in PSCs. The observed epigenetic remodeling supports a safe and improved neural fate.

### 2.7 Cells Undergo Metabolic Reprogramming During the Transition from PSCs to NSCs

**FIGURE 3:**
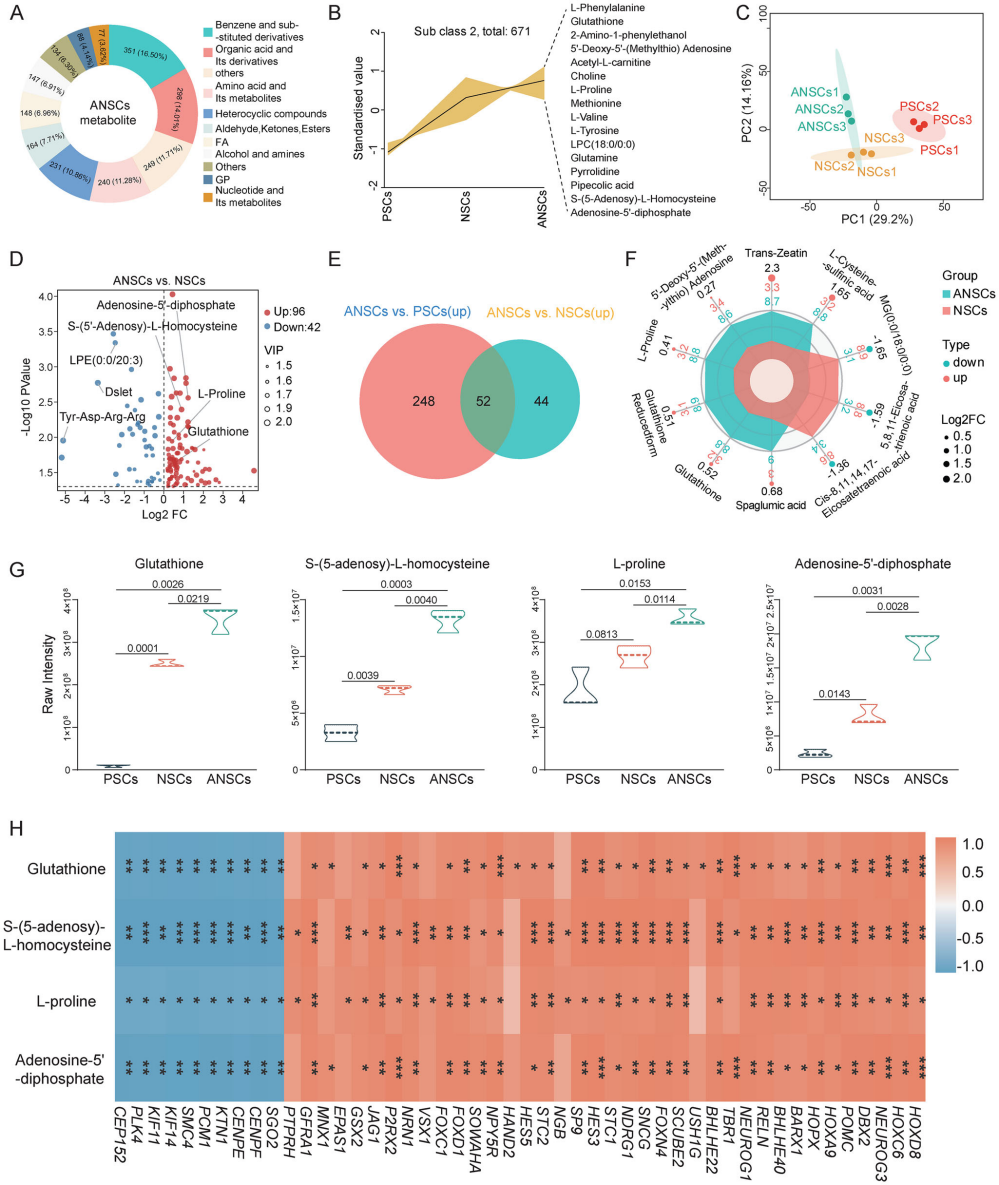
The transition from PSCs to ANSCs involves metabolic reprogramming. (A), Metabolite class distribution identified in ANSCs. Pie chart illustrating the proportion of per class. (B), K‐means clustering of differentially abundant metabolites across PSCs, NSCs, and ANSCs. (C), Principal component analysis (PCA) of metabolites from PSCs, NSCs, and ANSCs. (D), Volcano plot showing differential metabolite abundance (ANSCs vs. NSCs, |log_2_FC| > 1, *P* < 0.05). (E), Venn diagram showing metabolites both upregulated in ANSCs vs. PSCs and ANSCs vs. NSCs. (F), Radar chart showing the top 10 metabolites with the highest qualitative scores in ANSCs and NSCs. (G), Violin plots showing the relative abundance (raw peak area) of four key metabolites in PSCs, NSCs, and ANSCs. *P* values were determined using two‐tailed Student's *t*‐tests. (H), Heatmap showing Pearson correlations between metabolite abundance and neuroectodermal gene expression (Z‐scores). Red shows positive correlations. Blue shows negative correlations. ^*^ *P* < 0.05, ^**^ *P* < 0.01, ^***^ *P* < 0.001.

To investigate the metabolic features of ANSCs, we conducted comprehensive metabolomic profiling of ANSCs, NSCs, and PSCs. Our data showed that ANSCs metabolites were mainly enriched in benzene and substituted derivatives (16.5%), organic acids and derivatives (14.01%), amino acids and metabolites (11.28%), and heterocyclic compounds (10.86%) (Figure 3A). Although ANSCs, NSCs, and PSCs displayed similar metabolic profiles (Figure S8A), K‐means clustering analysis revealed distinct differences between these cell types. K‐means clustering revealed 671 metabolites (in subclass 2), exhibiting progressively increasing abundance from PSCs to NSCs to ANSCs (Figure 3B). Other metabolites did not exhibit a sustained increase throughout the induction process (Figure S8B).

Principal component analysis (PCA) revealed substantial differences between PSCs and both NSCs and ANSCs (Figure 3C). Volcano plot analysis revealed that, compared to NSCs, ANSCs exhibited 96 upregulated and 42 downregulated metabolites (Figure 3D), indicating that the isolated variation of TTNPB led to these metabolite discrepancies. Compared with PSCs, ANSCs exhibited 300 upregulated and 51 downregulated differential metabolites (Figure S8C). A total of 52 metabolites were upregulated in ANSCs compared to both PSCs and NSCs (Figure 3E), suggesting their potential roles in neural ectodermal lineage specification. The top 10 metabolites with the highest qualitative scores in ANSCs and NSCs are shown in Figure 3F. Differential heatmaps supported the strong metabolomic divergence between NSCs and ANSCs (Figure S8D), along with PSCs and ANSCs (Figure S8E).

### 2.8 ADP, SAH, Glutathione (GSH), and L‐Proline Promote Neural Fate Commitment in ANSCs

Next, by referencing the KEGG and HMDB databases, we identified disease associations for the differentially abundant metabolites. Notably, several upregulated metabolites in ANSCs, including adenosine 5′‐diphosphate (ADP), S‐(5′‐adenosyl)‐L‐homocysteine (SAH), GSH, and L‐Proline, were linked to neurological disorders. Given their established roles in neural pathology, we hypothesized that these metabolites may also contribute to neural development, prompting us to focus on these four key metabolites in subsequent analyses. To further validate these findings, we plotted violin plots based on the relative abundances (raw peak areas) of the four key metabolites. The results showed that ADP, SAH, GSH, and L‐Proline levels were significantly elevated in ANSCs compared to PSCs and NSCs (Figure 3G).

To explore potential functional associations, we performed Pearson correlation analysis between the key metabolites and gene expression profiles. Gene expression revealed that genes upregulated in ANSCs were associated with neural ectodermal identity and neural maturation, whereas genes upregulated in NSCs were related to cell cycle (Figure S8F). The four metabolites upregulated in ANSCs exhibited strong positive correlations with neural development‐related gene clusters and negative correlations with cell cycle‐associated genes (Figure 3H). Spearman's correlation further confirmed that ADP, SAH, GSH, and L‐proline were significantly positively correlated with key neural regulators such as *HOXA9*, *NEUROG1*, and *NEUROG3* (Figure S8G–J).

Metabolomic profiling of H9‐PSCs, H9‐NSCs, and H9‐ANSCs also revealed distinct metabolic signatures, as evidenced by clear separations in PCA, orthogonal partial least squares discriminant analysis (OPLS‐DA), hierarchical clustering, and K‐means analyses (Figure S9A–D). The key metabolites SAH, GSH, and L‐proline were significantly elevated in H9‐ANSCs (Figure S9E). Correlation analysis further demonstrated significant positive associations between these metabolites and neural regulatory factors (Figure S9F). These findings suggested that these metabolites might play important roles in promoting the neural ectodermal differentiation potential of ANSCs.

### 2.9 TTNPB‐SAH/PEMT‐Choline Metabolic Network Promotes Neuroectodermal Fate Specification

**FIGURE 4:**
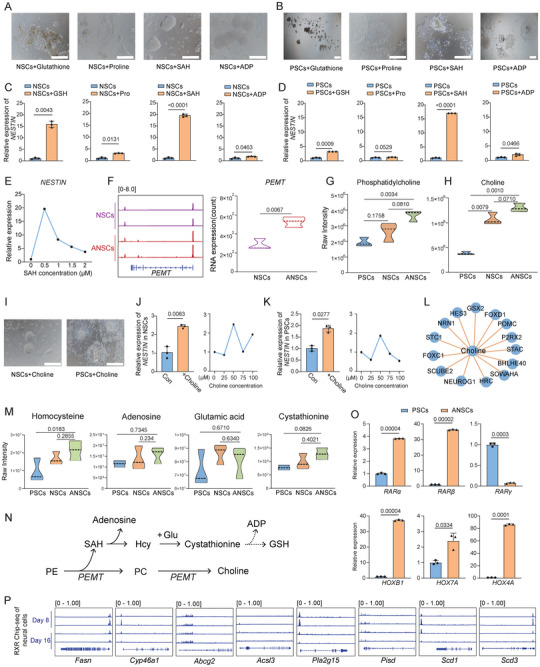
TTNPB influences neural differentiation through the PEMT/SHA/Choline core metabolic network. (A), Morphology of NSCs treated with individual metabolites. Scale bar: 100 µm. SAH: S‐(5′‐adenosyl)‐L‐homocysteine; ADP: adenosine 5′‐diphosphate. (B), Morphology of PSCs treated with individual metabolites. Scale bar: 100 µm. (C), RT‐qPCR analysis of the *NESTIN* expression of NSCs and NSCs treated with individual metabolites for 6 days. GSH: glutathione; Pro: L‐proline. Data were normalized to *GAPDH*. Error bars represent mean ± SD. (n = 3 biological replicates). *P* values were determined using two‐tailed Student's *t*‐tests. (D), RT‐qPCR analysis of the *NESTIN* expression of PSCs and PSCs treated with individual metabolites for 6 days. Data were normalized to *GAPDH*. Error bars represent mean ± SD. (n = 3 biological replicates). *P* values were determined using two‐tailed Student's *t*‐tests. (E), RT‐qPCR analysis of the *NESTIN* expression of PSCs treated with SAH in different concentrations. Data were normalized to *GAPDH*. Error bars represent mean ± SD. (n = 3 biological replicates). *P* values were determined using two‐tailed Student's *t*‐tests. (F), Left: IGV tracks showing ATAC‐seq signals near the *PEMT* locus. Right: RNA‐seq analysis of *PEMT* expression in NSCs and ANSCs. *P* values were determined using two‐tailed Student's *t*‐tests. (G, H), Violin plots showing the relative abundance (raw peak area) of phosphatidylcholine (G) and choline (H) in PSCs, NSCs, and ANSCs. *P* values were determined using two‐tailed Student's *t*‐tests. (I), Morphology of NSCs and PSCs treated with choline for 6 days. Scale bar: 100 µm. (J), Left: RT‐qPCR analysis of the *NESTIN* expression in NSCs and NSCs treated with choline for 6 days. Right: expression of *NESTIN* in NSCs treated with choline in different concentrations. Error bars represent mean ± SD. (n = 3 biological replicates). *P* values were determined using two‐tailed Student's *t*‐tests. (K), Left: RT‐qPCR analysis of the *NESTIN* expression in PSCs and PSCs treated with choline for 6 days. Right: Expression of *NESTIN* of PSCs treated with choline in different concentrations. Error bars represent mean ± SD. (n = 3 biological replicates). *P* values were determined using two‐tailed Student's *t*‐tests. (L), Network of Pearson correlations between choline and neural‐associated genes. Yellow lines: positive correlations. (M), Violin plots showing the relative abundance (raw peak area) of homocysteine, adenosine, glutamic acid, and cystathionine in PSCs, NSCs, and ANSCs. Error bars represent mean ± SD. (n = 3 biological replicates). *P* values were determined using two‐tailed Student's *t*‐tests. (N), Schematic diagram illustrating the metabolic process. Glu: glutamic acid; Hcy: homocysteine; PC: phosphatidylcholine; PE: phosphatidylethanolamine. (O), RT‐qPCR analysis of retinoic acid (RA) signaling genes expression in PSCs and ANSCs. Data were normalized to *GAPDH*. Error bars represent mean ± SD. (n = 3 biological replicates). *P* values were determined using two‐tailed Student's *t*‐tests. (P), IGV snapshot of RXR ChIP‐seq data reanalyzed from Zoltan Simandi's study (ref [12]) (GSE101768), showing RXR binding profiles at day 8 and day 16 of neural differentiation.

To further investigate whether ADP, SAH, GSH, and L‐proline could facilitate neuroectodermal fate specification, we individually supplemented NSCs and PSCs culture media with these metabolites. We observed that GSH induced partial cell death in NSCs and caused morphological changes from floating spheres to more adherent cells (Figure 4A). A similar death phenomenon was observed in PSCs upon GSH treatment, whereas L‐proline and ADP had minimal morphological impact. Notably, SAH treatment led to the emergence of neurosphere‐like structures in PSCs (Figure 4B).

To evaluate the effect of metabolite treatment on cells, we assessed the expression levels of the neuroectodermal marker *NESTIN*. In NSCs, all four metabolites mentioned above significantly upregulated *NESTIN* expression, with SAH showing the strongest effect, inducing a more than 15‐fold increase (Figure 4C). In PSCs, ADP, SAH, and GSH also significantly enhanced *NESTIN* expression, with SAH demonstrating the most potent effect (Figure 4D). Based on the pronounced effect of SAH demonstrated above, we examined its effects at different concentrations. The data indicated that 0.5 µm SAH yielded the highest efficacy, whereas increasing the concentration resulted in diminished effects (Figure 4E).

Previous studies have shown that SAH is a byproduct of the PEMT (phosphatidylethanolamine N‐methyltransferase) pathway [37], which converts phosphatidylethanolamine (PE) to phosphatidylcholine (PC) through stepwise methylation. PE served as the initial substrate of this methylation process. The elevated SAH levels in ANSCs suggested robust activation of the PEMT pathway. Consistent with significantly high *PEMT* expression, ANSCs showed greater chromatin accessibility at the *PEMT* promoter (Figure 4F). Additionally, the PEMT pathway product PC was highly abundant in ANSCs (Figure 4G).

PC is one of the most abundant phospholipids in cells, playing crucial roles in membrane structure and signaling [41], and serves as an endogenous source of choline in humans [42]. Previous studies demonstrated that choline facilitates the differentiation of PSCs toward the neuroectodermal lineage [43, 44]. Consistent with these studies, our data showed that choline levels were highest in ANSCs and lowest in PSCs (Figure 4H). When choline was added to the culture medium of NSCs or PSCs, obvious morphological changes were observed in the colonies, indicative of differentiation (Figure 4I). Expanding on this observation, choline significantly upregulated *NESTIN* expression and promoted neural differentiation; however, this effect exhibited a dose‐dependent threshold, decreasing when concentrations exceeded 50 µm (Figure 4J,K). Moreover, Pearson correlation analysis also showed that choline levels were positively correlated with the expression of neuroectodermal genes, which further confirmed that choline enhanced neural differentiation (Figure 4L).

Given the elevated SAH levels in ANSCs and the need for intracellular balance [45], we analyzed the SAH hydrolysis products homocysteine (Hcy) and adenosine. Both showed the highest levels in ANSCs, indicating enhanced SAH catabolism (Figure 4M). Glutamic acid (Glu), another key precursor of GSH biosynthesis [46] and the intermediate metabolite cystathionine, also showed increased levels, suggesting enhanced GSH biosynthetic flux, which may have further elevated intracellular GSH and ADP levels (Figure 4M,N). In summary, choline and SAH, key metabolites involved in the PEMT pathway, as well as GSH and ADP, which are influenced by SAH catabolism, promoted neuroectoderm induction through a PE‐PC centered metabolic network (Figure 4N).

To further explore the potential connection between TTNPB and the metabolic network described above, we analyzed the expression of key genes involved in RA signaling. Our results showed that TTNPB treatment upregulated the expression of genes encoding RARs, RXRs, and downstream targets of RA signaling, including *HOXB1*, *HOXA7*, and *HOXA4* (Figure 4O). Given the evolutionary conservation of the RA signaling pathway in vertebrates [15, 30, 47, 48], we analyzed previously published datasets of neural cells derived from PSCs to identify genes directly regulated by the RAR/RXR heterodimer [12]. Our analysis revealed that RXR binds directly to and activates genes that are directly or indirectly involved in the metabolic conversion of PE to PC (Figure 4P; Figure S10A). These results support the hypothesis that TTNPB promotes neural differentiation by activating RA signaling, which in turn directly modulates cellular metabolic pathways. Together, our findings demonstrated that SAH, ADP, GSH, and choline, key metabolites generated during the metabolic reprogramming of the PSCs‐to‐ANSCs transition, as well as TTNPB‐regulated metabolites, promote neuroectoderm induction, highlighting the critical role of metabolism in cell‐fate determination.

### 2.10 ANSCs and NSCs Engraft in the Rat Hippocampus and Alleviate Depressive‐Like Behaviors

**FIGURE 5:**
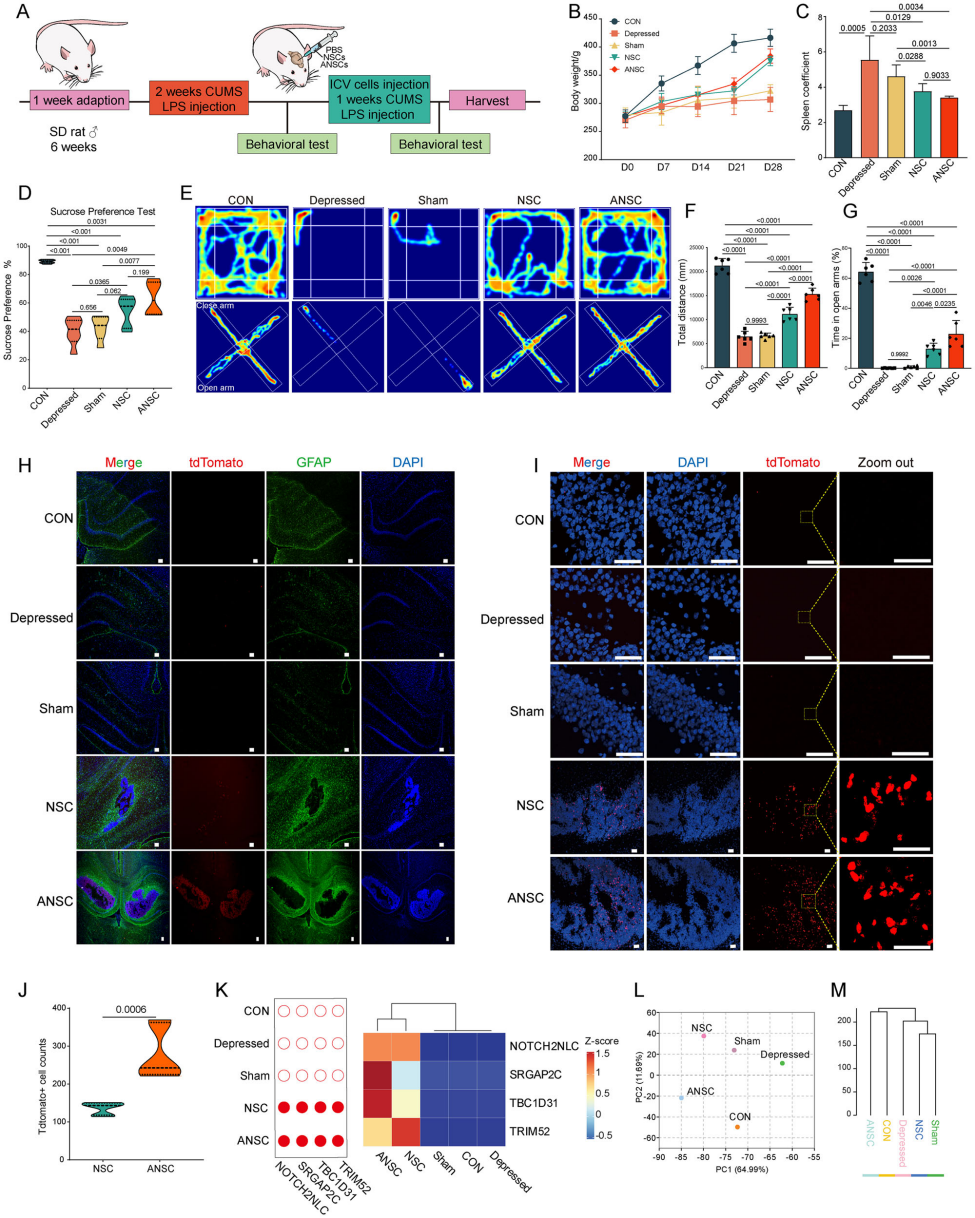
NSCs and ANSCs transplantation ameliorates depressive‐like behaviors and remodels hippocampal transcriptomes in LPS‐ and CUMS‐induced rat models. (A), Schematic illustration of the experimental design for the establishment of LPS‐ and CUMS‐induced depressive‐like rat models and stereotactic transplantation of NSCs or ANSCs into the hippocampus. (B), Body weight (BW) was measured weekly in rats from each group (n = 6). *P* values were determined using Welch's *t*‐tests. (C), Spleen coefficient of rats from each group at the experimental endpoint (day 28, n = 6). *P* values were determined using Welch's *t*‐tests. (D), Sucrose preference test (SPT) assessing sucrose consumption in rats from each group before and after PBS, NSCs, or ANSCs injection (n = 6). *P* values were determined using Welch's *t*‐tests. (E), Open field test (OFT) and elevated plus maze (EPM) behaviors of rats were recorded before and after PBS, NSCs, or ANSCs injection and analyzed using an automated animal behavior analysis system. (F), Total distance traveled during the OFT in each group. *P* values were determined using Welch's *t*‐tests. (G), Percentage of time spent in the open arms of the EPM in each group. *P* values were determined using Welch's *t*‐tests. (H), Representative immunofluorescence micrographs of the rat hippocampus. Red: tdTomato, green: GFAP. Scale bar, 100 µm. (I), Representative immunofluorescence micrographs of the enlarged rat hippocampus. Nuclei were stained with DAPI (blue), and transplanted cells were identified by tdTomato (red). Scale bar, 50 µm. (J), The number of tdTomato^+^ cells with identifiable nuclear was quantified in representative microscopic fields of the NSC and ANSC group of rat hippocampal sections. (K), Detection of human‐derived transcripts in rat hippocampal RNA‐seq data using human‐specific gene sequences. Open circles indicate samples without detectable human‐specific sequences, whereas filled circles indicate samples with detectable human‐derived transcript fragments. Heatmap showing normalized read counts of detected human‐specific transcript fragments in rat hippocampal RNA‐seq data. (L), Principal component analysis (PCA) of hippocampal transcriptomes from CON, Depressed, Sham, NSC, and ANSC groups. (M), Hierarchical clustering of hippocampal transcriptomes from CON, Depressed, Sham, NSC, and ANSC groups (distance metric: 1‐Spearman correlation coefficient).

To assess the functional potential of PSCs‐derived ANSCs and NSCs, we conducted functional studies using a neurological disease model. Tdtomato‐labeled ANSCs and NSCs were stereotactically injected into the hippocampi of rats exposed to chronic unpredictable mild stress (CUMS) and lipopolysaccharide (LPS). Rat behaviors were then systematically assessed (Figure 5A). Exposure to CUMS and LPS led to a marked reduction in body weight in both the depressed and sham groups, whereas rats receiving ANSCs or NSCs transplantation exhibited significantly less weight loss (Figure 5B). The spleen and liver index, which reflects the level of systemic inflammation, also decreased following ANSCs or NSCs transplantation (Figure 5C; Figure S10B).

The sucrose preference test revealed that sucrose consumption was significantly decreased in both the depressed and sham groups compared with the control group, whereas transplantation of ANSCs or NSCs partially restored sucrose preference (Figure 5D). To further assess depressive‐like behaviors, additional behavioral assays, including the open field and elevated plus maze tests, were performed. In the open field test, both the total locomotor distance and time spent in the central area were significantly decreased in the depressed and sham groups. In contrast, transplantation of ANSCs and NSCs increased the total locomotor distance and duration of central area occupancy relative to the depressed and sham groups (Figure 5E,F; Figure S10C). Similarly, in the elevated plus maze test, compared with the control group, the time spent in the open arms and the frequency of open arm entries were significantly reduced in the depressed and sham groups; In contrast, ANSCs or NSCs transplantation increased these two indices relative to the depressed and sham groups. (Figure 5E,G; Figure S10D).

To verify whether ANSCs and NSCs could survive and engraft in the rat hippocampus following transplantation, we performed immunofluorescence assays on hippocampal tissues seven days post‐transplantation. We observed that tdTomato‐labeled ANSCs and NSCs localized in the rat hippocampus region (GFAP+) (Figure 5H). These cells displayed intact nuclear morphology and robust tdTomato expression (Figure 5I). Compared with rats receiving NSCs transplantation, a greater number of tdTomato‐positive cells were detected in the hippocampal region of rats transplanted with ANSCs (Figure 5J). For further validation of this finding, we selected four human‐specific gene sequences and aligned them against the raw RNA‐sequencing data from rat hippocampal samples. Human‐derived sequences were detected exclusively in the ANSCs‐ and NSCs‐transplanted groups, but were absent in the depressed, sham, and control group (Figure 5K). As expected, more counts were detected in the ANSCs group than in the NSCs group, implying a better functional potential of ANSCs (Figure 5K). In line with this enhanced graft persistence, RNA sequencing of hippocampal tissues demonstrated that PCA and hierarchical clustering positioned the ANSCs‐transplanted group closer to the control group, suggesting that ANSCs transplantation more effectively restored the hippocampal transcriptional landscape toward a physiological state (Figure 5L,M). Together, these results indicated that ANSCs and NSCs were successfully engrafted into the rat hippocampus and alleviated depressive‐like behaviors in neurological disease models. These observations highlight the potential use of human PSCs‐derived neuronal cells for regenerative therapies targeting neurological disorders.

## 3 Conclusion

**FIGURE 6:**
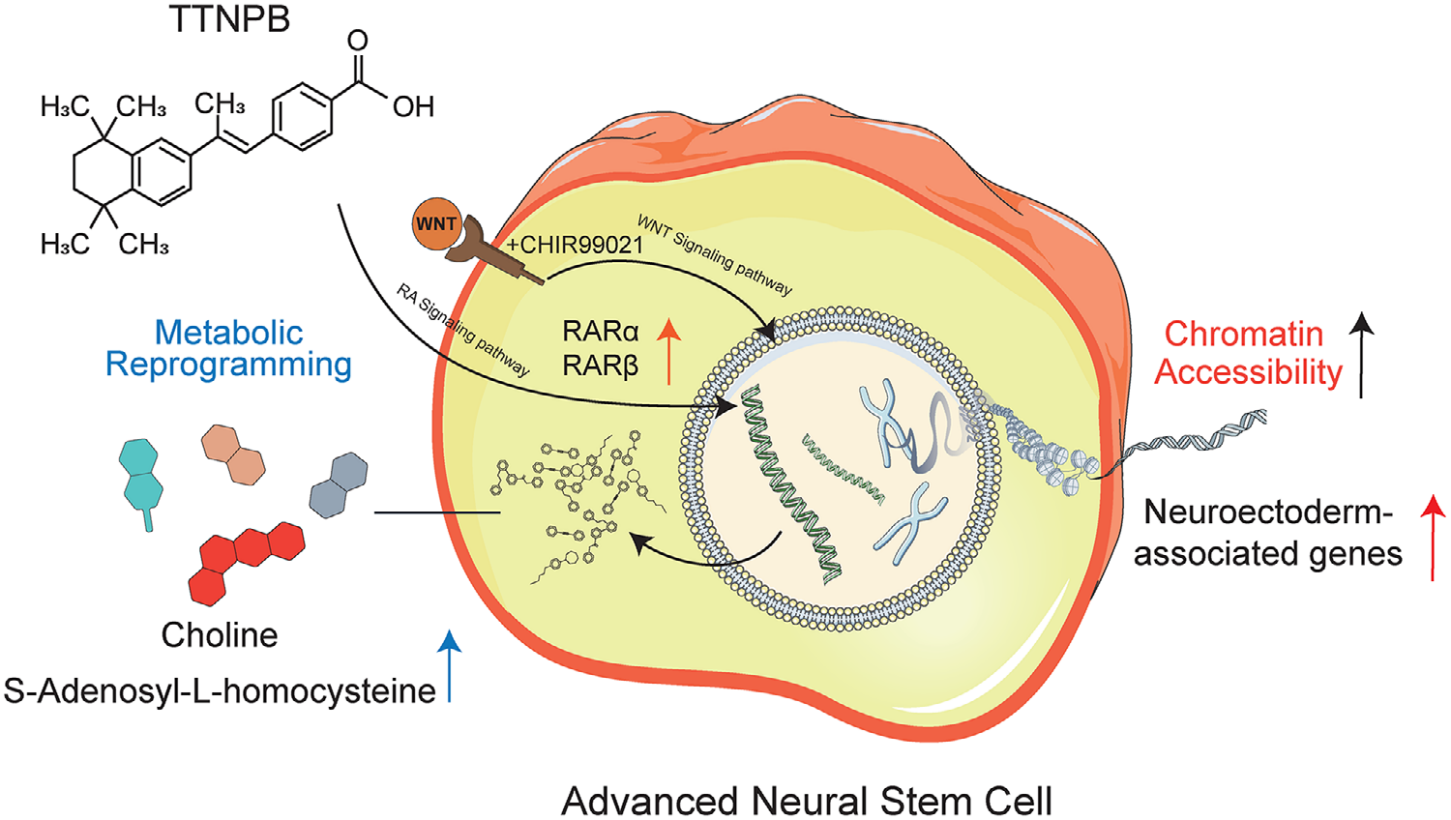
Proposed molecular pathway by which TTNPB promotes neural ectoderm fate through chromatin remodeling and metabolic reprogramming. RA: Retinoic Acid; RARα, Retinoic Acid Receptor alpha; RARβ, Retinoic Acid Receptor beta.

In this study, we established a chemically defined and efficient strategy to generate an advanced NSCs state from human PSCs using the RA receptor agonist TTNPB and the Wnt activator CHIR99021. Compared with NSCs, ANSCs exhibited enhanced neuroectodermal identity and robust neural lineage commitment while maintaining long‐term self‐renewal. Mechanistically, TTNPB amplified CHIR99021‐driven neural induction by engaging RA signaling and reshaping the transcriptional and epigenetic landscape (Figure 6). Functionally, ANSCs displayed superior neural differentiation in vitro and improved survival, engraftment, and in vivo functionality. Taken together, these findings highlighted TTNPB as a central regulator that coordinates signaling, chromatin accessibility, and metabolism to drive stable neuroectodermal fate specification.

## 4 Discussion

With the advancement of chemical reprogramming in stem cell research, the use of small‐molecule combinations has emerged as a prominent and rapidly evolving area of investigation. These combinations can be applied to re‐establish totipotency [31], pluripotency [33], or to drive directed lineage differentiation [49]. In this study, we established chemically defined PSCs as advanced NSCs by synergistically applying CHIR99021, a Wnt signaling activator, and TTNPB, a potent RAR agonist. In a previous study, Ding et al. used TTNPB to achieve a totipotent state [31]. Our data revealed that TTNPB combined with CHIR99021 not only recapitulated key molecular and phenotypic features of neural ectoderm commitment. It also generated a distinct neural‐primed population with enhanced lineage‐specific gene expression and a characteristic epigenetic landscape.

Previous protocols for neural induction have often relied on complex mixtures of growth factors or poorly defined culture systems, which compromise reproducibility and mechanistic interpretation [50, 51]. In contrast, the use of CHIR99021 and TTNPB in the N2B27 medium represents a minimal yet sufficient induction system. We demonstrated that even at low concentrations, TTNPB significantly upregulated neuroectodermal transcription factors in the absence of notable cytotoxicity or morphological anomalies. Previous studies have demonstrated that activation of RA and WNT signaling pathways promotes mesodermal differentiation of stem cells, leading to the induction of chondrocytes [52], sinus node‐like cells, and activation of hair follicle cells [53, 54]. Expanding upon these findings, our study primarily focused on the ectodermal lineage, underscoring the potent neural‐inductive role of RAR signaling in synergy with Wnt activation. This likely mimics early embryonic patterning signals, where RA and Wnt gradients specify neuroectoderm fate [55]. In our culture system, TTNPB activates the RA signaling pathway primarily through the upregulation of *RARα* and *RARβ*, but not *RARγ*, which differs from previous studies [56].  This suggests that TTNPB may engage distinct activation mechanisms depending on the specific culture context.

Compared to NSCs, ANSCs preferentially differentiated into neuroectodermal lineages, which is consistent with their significantly heightened expression of key neuroectodermal regulators. Furthermore, the elevated expression of astrocytes and neural progenitor markers such as *GFAP* and *TUBB3* in ANSCs reflects their readiness for lineage specification. These characteristics make ANSCs less suitable for pluripotency, but highly advantageous for lineage‐specific disease modeling, drug screening, and regenerative strategies targeting the nervous system.

Moreover, we propose for the first time that TTNPB enhances the effect of CHIR99021 through chromatin remodeling. Our findings support a model in which TTNPB acts as a chromatin remodeling enhancer, facilitating the neural induction potential of CHIR99021 by increasing chromatin accessibility at neuroectodermal loci. A progressive increase in chromatin openness from PSCs to NSCs to ANSCs, particularly in regions surrounding transcription start sites, coincided with the transcriptional upregulation of neuroectodermal genes. PSCs experienced an epigenetic remodeling characterized by the loss of pluripotency and a shift toward neural specification driven by TTNPB (Figure 6). Surprisingly, no evidence of DNA damage or metabolic dysregulation was observed despite increased global chromatin accessibility [57], demonstrating that TTNPB, in cooperation with our induction system, is safe and reliable. Overall, elucidating how TTNPB orchestrates chromatin remodeling in combination with cytokines or small‐molecule cues to reconfigure cellular plasticity, whether by inducing totipotency, reinstating pluripotency, or directing precise lineage differentiation, represents a transformative frontier in reprogramming biology.

Metabolic activity, which is indicative of cellular demand, is emerging as a key factor in neural cell‐fate decisions [58]. Our metabolomics data showed that ANSCs displayed a distinct metabolic signature compared to both PSCs and NSCs, suggesting that metabolic reprogramming is closely tied to lineage specification. Among the significantly altered metabolites, SAH, ADP, GSH, and L‐proline were not only affected by the culture system, but also enhanced neuroectoderm fate commitments. Exhibiting the most potent neuroectoderm‐inductive effects, SAH is a byproduct of the PEMT pathway that promotes phosphatidylcholine synthesis. Subsequently, phosphatidylcholine is metabolized into choline, which serves as an important endogenous source of this metabolite in humans [59]. Whether DNA methylation levels in ANSCs are altered due to the high expression of SAH and whether SAH regulates cell fate by affecting DNA methylation requires further experimental validation.

The levels of the SAH catabolic products, homocysteine and adenosine, were also elevated in ANSCs, indicating active SAH turnover. Homocysteine feeds into the GSH synthesis pathway, and we observed upregulation of related intermediates (cystathionine, glutamate), suggesting a metabolic axis from SAH to GSH and ADP (Figure 4N) [60]. This axis may provide redox and energetic support for neural differentiation. Meanwhile, the PEMT pathway also generates choline. When supplemented exogenously, choline enhanced neural gene expression and cell differentiation via the PEMT pathway, confirming its role in membrane biosynthesis and neural development. Pearson correlation further linked choline abundance with neuroectoderm‐associated genes such as *NEUROG1* (Figure 4L), supporting the hypothesis that choline availability is critical for neural fate commitment [61, 62]. These evidences support a model wherein extrinsic signaling, epigenetic state, and intrinsic metabolic cues cooperatively guide cell fate decisions.

Hippocampal transplantation of human ANSCs and NSCs partially improved depressive‐like behaviors induced by CUMS and LPS in rats, likely reflecting indirect modulation of the hippocampal microenvironment rather than direct neuronal replacement. These benefits are consistent with established mechanisms of NSCs/NPCs, including the reduction of oxidative stress and neuroinflammation, modulation of key signaling pathways such as WNT [63], secretion of neurotrophic factors (e.g., BDNF, GDNF, NGF) [64], and support of endogenous neural cell proliferation and circuit reconstruction [1, 65]. These findings demonstrate that stem cell‐based approaches can modulate both the cellular and molecular components of the injured hippocampus, highlighting the feasibility and therapeutic promise of ANSCs and NSCs in neuropsychiatric disease models.

In summary, we demonstrated that TTNPB serves as a central regulator that coordinates signaling, chromatin accessibility, and metabolism to drive robust and stable neuroectodermal fate specification. Using TTNPB in combination with CHIR99021, we efficiently generated ANSCs from human PSCs, which exhibited enhanced neuroectodermal differentiation potential and long‐term self‐renewal. Hippocampal transplantation of ANSCs further confirmed their functional efficacy in vivo, highlighting their potential for mechanistic studies and future therapeutic applications in neurodevelopmental and neuropsychiatric disorders.

## 5 Experimental Section

### 5.1 Animal and Cell Lines

The animal studies were approved by the Institutional Animal Care and Use Committee at Inner Mongolia University NMGDX (Wu) 2022–0003. Six‐week‐old, male Sprague–Dawley (SD) rats (160–180 g) were purchased from Beijing Vital River Laboratory Animal Technology Co., Ltd. (Beijing, China). Animals were housed in a temperature‐controlled environment (22°C) under a 12‐h light/dark cycle, with ad libitum access to food and water. Rats were acclimated to the housing conditions for 1 week prior to the initiation of experiments.

The human PSCs line H9 (CSTR:19375.09.3101HUMSCSP302; RRID:CVCL_9773) was purchased from the China Center for Type Culture Collection. The human PSCs line W24 was obtained from the Wellcome Trust/Cancer Research UK Gurdon Institute, University of Cambridge (RRID not available). The iPSCs line Z1 (Z1‐IPS) was purchased from the Hunan Fenghui Biotechnology Co., Ltd. All cell lines used in this study were routinely tested and confirmed to be free of mycoplasma contamination.

### 5.2 Culture of Human Pluripotent Stem Cells

The H9, W24, and Z1 cells were cultured in mTeSR1 (StemCell Technologies, #85850) on plates that were pre‐coated with human vitronectin (Gibco, #A14700). W24 cells were passaged by at a 1:5 ratio with 0.02% EDTA (Sigma, # E8008) prepared by in Dulbecco's phosphate‐buffered saline. The 0.2 g EDTA was dissolved in 1 Liter Dulbecco's phosphate‐buffered saline (DPBS, Biological industries, #02‐020‐1A). H9 cells and Z1 cells were passaged by TrypLE (Gibco, #12604021). During passaging, all PSCs were cultured in mTeSR medium supplemented with Y‐27632 for 24 h (10 µm, Selleck, #S1049).

### 5.3 Neural Stem Cells Induction and Maintenance

Upon reaching 70%–80% confluency in a 24‐well plate, the PSCs (H9, W24 and Z1) were transitioned from the mTeSR1 culture medium to a neural induction medium referred to as CL medium [N2B27 medium supplemented with CHIR99021 (3 µm, Miltenyi Biotech, # 501897016) and human leukemia inhibitory factor (10 ng/mL, Millipore, LIF1010)] [11]. Cells were maintained under these conditions for more than 10 passages. Subsequently, the cells were transferred to an advanced neural induction medium, designated as CT medium. This CT medium, which consisted of N2B27 medium supplemented with CHIR99021 (3 µm, Miltenyi Biotech, # 501897016) and TTNPB (0.5 µm, Selleck, # S4627). The N2B27 medium: was prepared as 1:1 mixture of DMEM/F12 medium (Gibco, # 10565018) and neurobasal medium (Gibco, #21103049), supplemented with 0.5% N2 (Gibco, # 17502048), 1% B27 (Gibco, # 17504044), 1% Glutamax (Gibco, #35050061), 1% non‐essential amino acids (Gibco, #11140050), 100 µM β‐mercaptoethanol (Sigma, #M3148), 50ug/ml BSA‐Fraction V (Gibco, #15260‐037), and 1% penicillin‐streptomycin (Sigma, #P4333). Half volume of CT medium was replaced daily. The ANSCs derived from these hPSCs were maintained in CT medium and could be stably propagated over 50 passages. Unless otherwise specified in this article, all data pertaining to NSCs and ANSCs were derived from the W24 cell line.

### 5.4 Differentiation of Neural Stem Cells

Six to seven days after plating, ANSCs derived from NSCs (Passage 15) were dissociated into a single‐cell suspension using Accutase (Invitrogen, #A1110501), washed with DPBS, and resuspended in M10 medium [DMEM medium (Gibco), 10% FBS (Gibco), 1% penicillin‐streptomycin (Sigma)]. Cells were then seeded on vitronectin‐coated round glass coverslips in 24‐well plates and cultured in M10 medium supplemented with 5% FBS for differentiation over a period of 6–9 days, with the medium replacement every two days. Differentiation of cranial placode (CP) cells was performed according to a previously reported protocol [32].

### 5.5 Exogenous Metabolite Supplementation in PSCs and NSCs

Cells were washed with DPBS and resuspended in either PSCs or CL medium supplemented individually with the following exogenous metabolites: 10  mm glutathione (Aladdin, #G105427), 1 mm L‐proline (Sigma–Aldrich, #P6072), 150  µm Adenosine 5’‐diphosphate (ADP) (Aladdin, #A119474), 0.5  µm S‐(5’‐adenosyl)‐L‐homocysteine (SAH) (Sigma–Aldrich, #S7868) or 50 µm choline (Aladdin, #C875195). Cells were cultured under these conditions for six consecutive days, with daily medium replacement. At the end of the treatment period, *NESTIN* expression was assessed by RT‐qPCR to evaluate the effects of individual metabolites.

### 5.6 Microinjection of PSCs and ANSCs into Mouse 8‐Cell Stage Embryos and In Vitro Culture

Equivalent numbers of pluripotent stem cells and advanced neural stem cells were microinjected into mouse 8‐cell stage embryos using a piezo‐assisted micromanipulation system under a stereomicroscope. Following microinjection, the embryos were cultured in KSOM medium supplemented with CHIR99021, LIF, and TTNPB under mineral oil at 37°C in a 5% CO_2_ incubator. Embryos were monitored and collected after 24 and 48 h of in vitro culture to assess cell integration, proliferation, and lineage contribution. Embryonic development and the spatial distribution of injected cells were evaluated by immunofluorescence staining and confocal microscopy.

### 5.7 Alkaline Phosphatase Staining

Cells cultured in 24‐well plates were fixed with 4% paraformaldehyde (Solarbio, #P1110). Alkaline phosphatase activity was assessed to evaluate stem cell identity using a Leukocyte Alkaline Phosphatase Kits (Sigma, #86C‐1KT), following the staining procedure as outlined in the manufacturer's protocols.

### 5.8 Measurement of Cell Metabolites

Untargeted metabolomic profiling of human cell PSCs, NSCs, and ANSCs samples (n = 3 for each group) was performed by Wuhan Mteware Metabolic Biotechnology Co., Ltd. All samples were analyzed by liquid chromatography‐mass spectrometry (LC/MS) under both positive and negative ionization conditions. In the positive ion mode, separation was achieved with a Waters ACQUITY Premier HSS T3 Column (1.8 µm, 2.1 mm × 100 mm) using a gradient elution consisting of 0.1% formic acid in water (solvent A) and 0.1% formic acid in acetonitrile (solvent B). The gradient profile was as follows: 5% solvent B to 20% over 2 min, 20%–60% over 3 min, 60%–99% over 1 min, held at 99% for 1.5 min, then reverted to 5% solvent B within 0.1 min and equilibrated for 2.4 min. The column temperature was maintained at 40°C, with a flow rate of 0.4 mL/min and an injection volume of 4 µL.

Data acquisition was conducted in information‐dependent acquisition (IDA) mode utilizing Analyst TF 1.7.1 software (Sciex, Concord, ON, Canada). The source parameters were configured as follows: ion source gas 1 (GAS1) and ion source gas 2 (GAS2) were both maintained at 50 psi; the curtain gas (CUR) was set at 25 psi; the temperature (TEM) was held at 550°C; the declustering potential (DP) was adjusted to 60 V in positive mode and −60 V in negative mode; and the ion spray voltage floating (ISVF) was set to 5000 V in positive mode and −4000 V in negative mode. During the TOF MS scan, a mass range was set to 50–1000 Da, with an accumulation time of 200 ms, and the activation of dynamic background was enabled.

For the product ion scan, the parameters were as follows: a mass range of 25–1000 Da, an accumulation time of 40 ms, a collision energy of 30 V in positive mode and −30 V in negative mode, a collision energy spread of 15 V, unit resolution, a charge state of 1, an intensity threshold of 100 cps, isotopes exclusion within 4 Da, mass tolerance of 50 ppm, and monitoring of a maximum of 18 candidate ions per cycle. The resulting data were analyzed using the Maiwei Cloud platform (www.cloud.metware.cn).

### 5.9 Immunofluorescence of Cells

Cells were cultured on 8‐well chambered cell culture slides and then fixed using 4% paraformaldehyde (Solarbio, #P1110) for 20 min after three brief washes with DPBS. For cell permeabilization and blocking, cells were incubated in DPBS containing 1% BSA (Biological industries) and 0.1% Triton X‐100 (Sigma) for 30 min. Cells were incubated overnight at 4°C with primary antibodies diluted in the aforementioned buffer. After three washes with DPBS (10 min each), cells were then incubated with the secondary antibody for 1 h at room temperature, shielded from light exposure. Following an additional three washes (5 min each), the slides were mounted with a Vectashield mounting medium containing DAPI (Vector Laboratories, #H‐1200‐10). The slides were mounted with coverslips and sealed with nail polish, followed by imaging using a Nikon confocal microscope (Nikon Instruments, CO, Tokyo Japan). The primary antibodies used included: rabbit polyclonal OCT4 (Novus Biologicals, #NBP2‐15053, 1:200), rabbit polyclonal NANOG (PeproTech, #P236, 1:200), goat polyclonal SOX2 (R&D Systems, #AF2018, 1:200), rabbit monoclonal NESTIN (Boster, #PB9874, 1:200), rabbit polyclonal PAX6 (Elabscience, #E‐AB‐61653, 1:200), rabbit polyclonal N‐Cadherin (Abcam, #ab76057, 1:200), goat polyclonal Brachyury (R&D Systems, #AF2085, 1:100), goat polyclonal SOX17 (R&D Systems, #AF1924, 1:200), mouse monoclonal NeuN (CST, #93972, 1:100), mouse monoclonal TUBB3 (Bioss, #BSM‐33177M, 1:200), rabbit polyclonal GFAP (Bioss, #bs‐0199R, 1:200), and rabbit monoclonal CDX2 (Biogenex, #Cdx2‐88). The secondary antibodies used were Alexa Fluor 488 (Thermo Fisher Scientific, #A30629, 1:500) or Alexa Fluor 568 (Thermo Fisher Scientific, #A20103, 1:500).

### 5.10 Quantitative Real‐Time Polymerase Chain Reaction (qPCR)

Total RNA was extracted utilizing the RNeasy Plus Mini Kit (QIAGEN, #74134) in accordance with the manufacturer's instructions. First‐strand cDNA was synthesized using the Reverse Transcription System (Promega, #A3800). RT‐qPCR was subsequently performed with SYBR FAST (Kapa Biosystems, #KK4600) on a LightCycler 96 Instrument II (Roche Life Science, Mannheim, Germany) following the manufacturer's guidelines. The list of primer sequences is provided in Table S1.

### 5.11 RNA‐seq and Analysis

Total RNA was isolated from cultured cells or hippocampus of rats using the Trizol reagent kit (Invitrogen, #15596‐018) according to the manufacturer's instructions. The quality of RNA was evaluated, and the enriched mRNA was subsequently reverse‐transcribed into cDNA using random primers, followed by second‐strand cDNA synthesis. The resulting cDNA fragments were purified and sequenced via the Illumina HiSeq2500 platform (Illumina), facilitated by Gene Denovo Biotechnology.

Gene expression levels were quantified by calculating transcripts per million (TPM) for each gene. Differentially expressed genes were identified using the DESeq2 software. Genes exhibiting a false discovery rate (FDR) < 0.05 and an absolute fold change ≥ 2 were considered to be differentially expressed. Gene Ontology (GO) term enrichment analysis, KEGG pathway enrichment analysis, PCA and gene set enrichment analysis (GSEA) were performed using the OmicShare tools dedicated to data analysis (www.omicshare.com). Batch effects across different RNA‐seq datasets were corrected utilizing the limma R package. Heatmaps were generated using the pheatmap R package (v1.0.12) and Graphpad prism (v9.0). Interaction networks of the selected genes, along with the identified pathways and GO terms were constructed using the Cytoscape plugins ClueGO and CluePedia (v2.5.7).

### 5.12 ATAC‐seq Library Construction and Sequencing

ATAC‐seq protocol was executed in accordance with previously established methods with minor modifications [66]. Briefly, for each replicate, 1 × 10^5^ cells were lysed in a cold lysis buffer to isolate nuclei. Nuclei were immediately subjected to the Tn5 transposase reaction mix containing Tagment DNA Enzyme 1 (Illumina, #20034198) and incubated for 30 min at 37°C. Following transposition, DNA fragments were purified using the MinElute PCR Purification Kit (Qiagen, #28006) and subsequently amplified via PCR for 11–13 cycles employing barcoded primers as described by Buenrostro et al. [66].

The resulting libraries were further purified with AMPure XP beads (Beckman Coulter, #A63881). Library quality was validated using an Agilent 2100 Bioanalyzer (Agilent Technologies, #G2939BA) before proceeding to next‐generation sequencing. Paired‐end 150‐bp sequencing was conducted with the DNBSEQ‐T7 (MGI Tech). Each ATAC‐seq sample type was analyzed in two replicates.

### 5.13 ATAC‐seq Data Processing and Peak Calling

Raw sequencing reads were processed with Trim‐galore (v0.4.0; default settings) to remove Illumina adapters and low‐quality bases. Trimmed reads were aligned to the human reference genome (GRCh38) using Bowtie2 (v2.4.5; –very‐sensitive –no‐mixed –no‐discordant ‐X 2000). The resulting BAM files underwent sequential filtering: (1) Removal of PCR duplicate (Picard MarkDuplicates). (2) Exclusion of mitochondrial reads and low‐quality alignments (samtools view ‐q 30). (3) Removal of ENCODE Blacklist Regions (v2; GRCh38) using bedtools intersect ‐v. (4) Merging of biological replicates followed by subsampling to equal depth (samtools view ‐s).

Alignment files (BAM format) were converted into CPM‐normalized bigWig tracks using bamCoverage (DeepTools) with 50‐bp bins and –normalizeUsing CPM. The output was then piped to the plotPCA function from DeepTools to generate the PCA plot, and the plotCorrelation function to calculate the Pearson correlation coefficient between biological replicates. To assess average chromatin accessibility across genomic intervals of interest, CPM normalized ATAC‐seq normalized signal (CPM in 50 bp bins) was processed using the DeepTools computeMatrix (in scale‐regions mode with option ‐skipZeros) and visualized with plotHeatmap. Chromatin accessibility peaks were called with MACS3 (‐q 0.01 –keep‐dup all). Genome‐wide chromatin accessibility profiles were visualized using Integrative Genomics Viewer (IGV v2.16.2) by loading bigWig files alongside genome annotations.

### 5.14 ATAC‐seq Peak: Comparison, Annotation, and Transcription Factor Foot Printing Analysis

To assess chromatin accessibility dynamics across different cellular conditions, ATAC‐seq data were processed and analyzed using a comprehensive pipeline. Peaks from distinct experimental groups were compared using the BEDtools intersect function (v2.30.0), with a minimum overlap of 1 bp to define shared peaks. Differential accessibility analysis was performed using the DiffBind R package (v3.10.1), incorporating biological replicates and applying DESeq2‐based normalization and statistical modeling to identify differentially accessible regions (FDR < 0.05).

Peak annotation was conducted using the ChIPseeker package (v1.34.1), based on the human genome assembly GRCh38. Peaks overlapping promoter regions (defined as 3 kb upstream of transcription start sites), exons, or introns were classified as genic, whereas all remaining peaks were defined as intergenic.

Transcription factor footprinting analysis was performed using the HINT module from the Regulatory Genomics Toolbox (RGT, v0.13.3). To improve footprint detection sensitivity, biological replicates were pooled within each experimental condition, and broad ATAC‐seq peaks were called using MACS3. Footprints were identified within ATAC‐seq peaks using rgt‐hint in ATAC‐seq mode with paired‐end settings, and subsequently matched to JASPAR motifs using rgt‐motifanalysis in matching mode. To compare transcription factor (TF) binding activities between groups, peaks from both conditions were merged using the BED tools merge to generate a unified peak set. Footprinting was conducted within this unified peak set using pooled ATAC‐seq signals from each group. Differences in TF binding activity were quantified with rgt‐hint in differential mode, providing binding activity scores and associated *p*‐values (two‐tailed *t*‐test) for each TF to indicate statistically significant differences in TF occupancy between conditions.

### 5.15 CUMS Model

Depression‐like behaviors were induced using a combined Lipopolysaccharides (LPS) and chronic unpredictable mild stress(CUMS) paradigm. Rats received daily intraperitoneal injection of LPS (0.5mg/kg; Escherichia coli O55:B5, Sigma–Aldrich, #L5418) at 9:00 a.m. daily for 2 consecutive weeks, as previously reported [67]. The procedure for CUMS follows a similar to that in the previous study [68, 69, 70]. Briefly, rats were randomly exposed to the following stimuli for 3 weeks: (1) deprivation of food and water for 24 h; (2) restraint stress for 6 h; (3) odor stimulation for 24 h; (4) wet pad for 24 h; (5) tail clipping for 60 s; (6) cold water swimming (4°C) for 5 min; (7) cage tilting (45°) for 6 h; (8) no padding for 24 h; (9) shake for 10 min; (10) strobe for 12 h; (11) reversal of light/dark cycle for 24 h; (12) white noise for 12 h. Rats in the control group were not exposed to the CUMS procedure, without being disturbed except for the necessary procedures.

### 5.16 Behavioral Measurements

All behavioral experiments were conducted in accordance with previously published protocols, including the sucrose preference test, OFT, and EPM. Specifically, the procedures, experimental design, apparatus settings, and data analysis methods for each behavioral test were performed as described in earlier studies, without major modifications [69].

### 5.17 Stereotactic Surgery and NSCs and ANSCs Injection

Adult male SD rats were anesthetized with 2.5% isoflurane for induction and maintained under 1.5% isoflurane during surgery. Animals were positioned in a stereotaxic device (RWD Life Science. Inc). For cell injection, 5 µL of the cell suspension (total 1 × 10^5^ cells) was injected bilaterally into the hippocampus (coordinates from bregma: AP, ‐3.0 mm; ML, ±1.13 mm; DV, ‐4.25 mm). Both NSCs and ANSCs were injected at a rate of 10 nL/s. The capillary was left in place for 10 min after injection and then slowly retracted to minimize reflux.

### 5.18 Human‐Specific Gene Sequences Aligned

Human‐specific gene sequences (Table S2) were aligned against raw RNA‐sequencing data of rat hippocampal samples. Reads that perfectly matched the corresponding human‐specific sequences were identified, and the number of such reads was recorded as the count.

### 5.19 Statistical Analyses

All the statistical analyses were conducted using GraphPad Prism (version 7.0), or the R package [71]. All the data are presented as the mean ± standard deviation (SD). Comparisons between two groups were conducted using a two‐tailed Student's *t*‐test. Comparisons between multiple groups were conducted using one‐way ANOVA. For behavioral data with unequal variances, Welch's *t*‐test was applied as appropriate. Statistical significance was determined as ^*^ *P* < 0.05; ^**^ *P* < 0.01; ^***^ *P* < 0.001.

[The human PSCs line H9 (CSTR:19375.09.3101HUMSCSP302; RRID:CVCL_9773) was purchased from the China Center for Type Culture Collection. The human PSCs line W24 was obtained from the Wellcome Trust/Cancer Research UK Gurdon Institute, University of Cambridge (RRID not available). The iPSCs line Z1 (Z1‐IPS) was purchased from the Hunan Fenghui Biotechnology Co., Ltd. The animal studies were approved by the Institutional Animal Care and Use Committee at Inner Mongolia University NMGDX (Wu) 2022‐0003.]

## Ethics Statement

The animal studies were approved by the Institutional Animal Care and Use Committee at Inner Mongolia University NMGDX (Wu) 2022‐0003.

## Conflicts of Interest

The authors declare no conflicts of interest.

## Supporting information




**Supporting File**: advs74319‐sup‐0001‐SuppMat.pdf.

## Data Availability

The datasets supporting the conclusions of this article are available in the NCBI Gene Expression Omnibus repository under accession number GSE301755 (RNA‐seq) and GSE301756 (ATAC‐seq). Our metabolome data has been uploaded to the NGDC (ngdc.cncb.ac.cn) database with PRJCA042636 and PRJCA005485. Other data that support the findings of this study are available from the corresponding author upon reasonable request.
