# Supplementary material for: TTNPB Promotes Human Pluripotent Stem Cell‐to‐Neural Stem Cell Transition via Modulation of Chromatin Accessibility and the S‐(5′‐adenosyl)‐L‐homocysteine/Choline Metabolic Network
Source: Adv Sci (Weinh). 2026 Feb 10;13(22):e15648. doi: 10.1002/advs.202515648 (PMC13088305; doi:10.1002/advs.202515648)
Supplement: Supplementary file 1 — Supporting File: advs74319‐sup‐0001‐SuppMat.pdf. [file ADVS-13-e15648-s001.pdf]

Supporting Information

TTNPB Promotes Human Pluripotent Stem Cell-to-Neural Stem Cell Transition via Modulation of Chromatin Accessibility and the S-(5′-adenosyl)-L-homocysteine/Choline Metabolic Network

Ruilin Du, Yudi Ren, Qiaoqiao Meng, Peng Wei, Ruyu Zhu, Junjie Bao, Ye Yang, Shuo Yan, Chaorong Yue, Xueying Zhu, Shuo Cao, Chunxia Hao, Wei Sun, Yongli Song, Xihe Li, Zhimin Wu*, Siqin Bao*, Yanglin Chen**

This PDF file includes:

Figs. S1 to S10

Tables S1 to S2

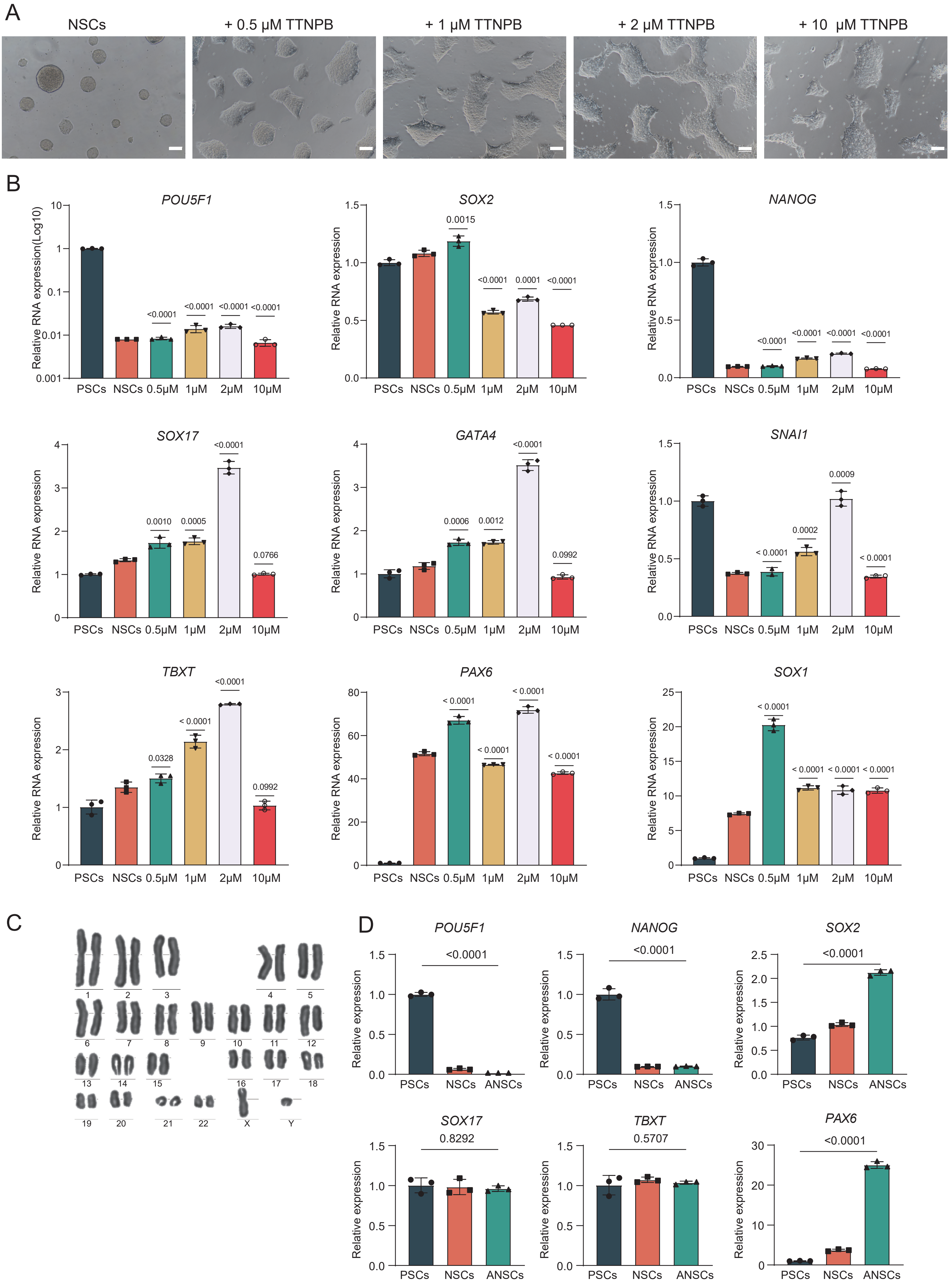
A
NSCs
+ 0.5 μM TTNPB
+ 1 μM TTNPB
+ 2 μM TTNPB
+ 10 μM TTNPB
B
POU5F1
Relative RNA expression(Log10)
10
1
0.1
0.01
0.001
<0.0001
<0.0001
<0.0001
<0.0001
SOX2
0.0015
<0.0001
0.0001
<0.0001
NANOG
<0.0001
<0.0001
<0.0001
<0.0001
Relative RNA expression
SOX17
<0.0001
0.0010
0.0005
0.0766
GATA4
<0.0001
0.0006
0.0012
0.0992
SNAI1
0.0009
0.0002
< 0.0001
< 0.0001
TBXT
<0.0001
< 0.0001
0.0328
0.0992
PAX6
< 0.0001
< 0.0001
< 0.0001
< 0.0001
SOX1
< 0.0001
< 0.0001
< 0.0001
< 0.0001
PSCs NSCs 0.5μM 1μM 2μM 10μM
C
1 2 3 4 5
6 7 8 9 10 11 12
13 14 15 16 17 18
19 20 21 22 X Y
D
POU5F1
<0.0001
NANOG
<0.0001
SOX2
<0.0001
SOX17
0.8292
TBXT
0.5707
PAX6
<0.0001
Relative expression
PSCs NSCs ANSCs

Fig. S1. Low concentrations of TTNPB are sufficient to promote neural differentiation.

(A), Morphology of NSCs treated with different concentrations of TTNPB. Scale bar: 100 μm. **(B),** RT-qPCR of pluripotency genes (*OCT4, SOX2, NANOG)*, endodermal and mesodermal genes (*SOX17, GATA4, SNAI1, TBXT*), and neuroectodermal genes (*PAX6, SOX1)* in PSCs, NSCs, and ANSCs treated with different concentrations of TTNPB. Data were normalized to *GAPDH*. Error bars represent mean ± SD. (n = 3 biological replicates). *P* values were determined using One-way ANOVA tests. **(C),** Representative karyotype of human ANSCs (passage 50) showing a normal diploid chromosomal complement (46, XY). **(D)**, RT-qPCR analysis of pluripotency (*POU5F1, NANOG, SOX2*) and lineage (*SOX17, TBXT, PAX6*) markers in PSCs, NSCs, and ANSCs. Data were normalized to *GAPDH*. Error bars represent mean ± SD. (n = 3 biological replicates). *P* values were determined using One-way ANOVA tests.

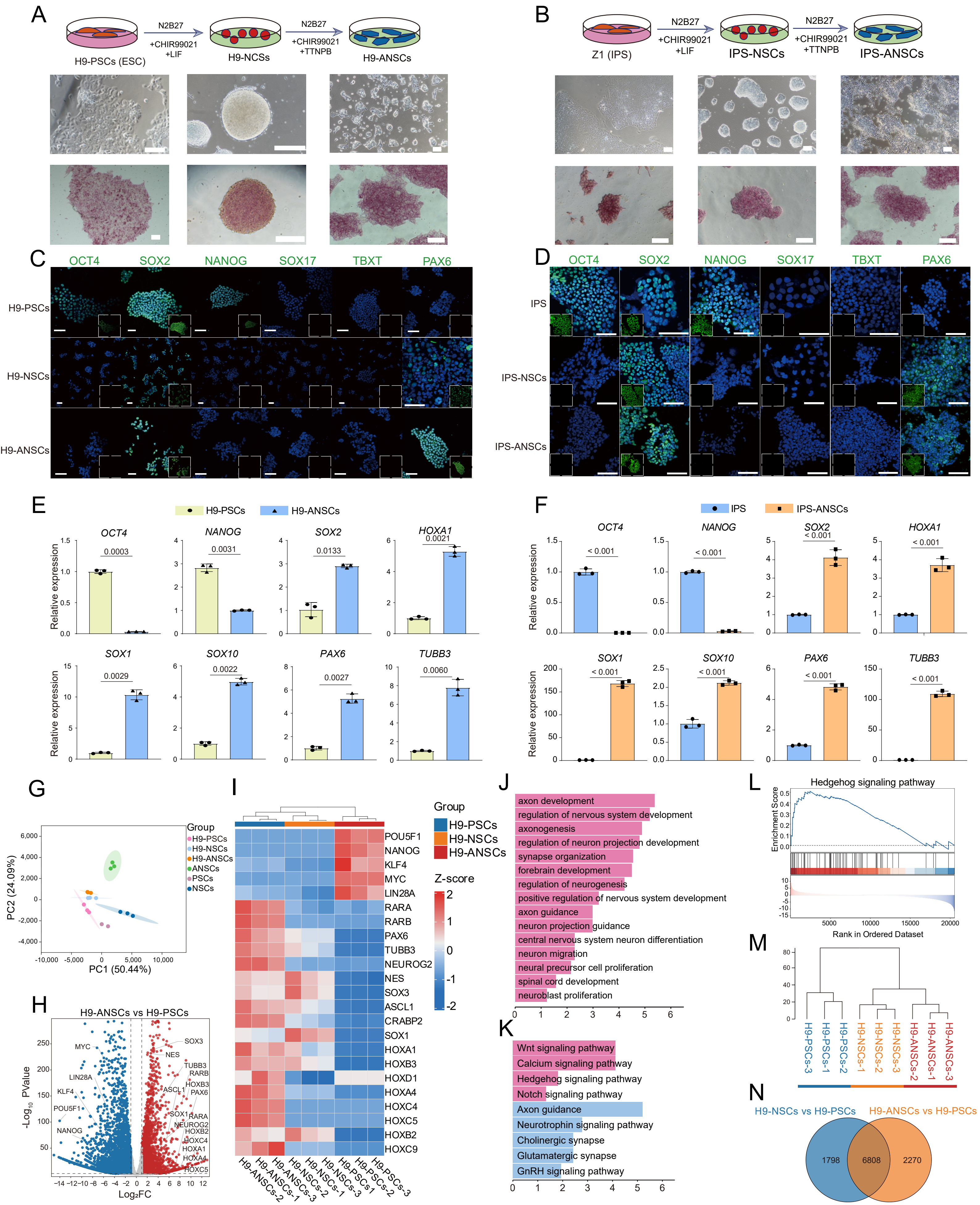
A
H9-PSCs (ESC)
N2B27 +CHIR99021 +LIF
H9-NCSs
N2B27 +CHIR99021 +TTNPB
H9-ANSCs
B
Z1 (IPS)
IPS-NSCs
IPS-ANSCs
C
OCT4 SOX2 NANOG SOX17 TBXT PAX6
H9-PSCs
H9-NSCs
H9-ANSCs
D
IPS
IPS-NSCs
IPS-ANSCs
E
H9-PSCs H9-ANSCs
Relative expression
OCT4 0.0003
NANOG 0.0031
SOX2 0.0133
HOXA1 0.0021
SOX1 0.0029
SOX10 0.0022
PAX6 0.0027
TUBB3 0.0060
F
IPS IPS-ANSCs
OCT4 < 0.001
NANOG < 0.001
SOX2 < 0.001
HOXA1 < 0.001
SOX1 < 0.001
SOX10 < 0.001
PAX6 < 0.001
TUBB3 < 0.001
G
Group
H9-PSCs
H9-NSCs
H9-ANSCs
ANSCs
PSCs
NSCs
PC2 (24.09%)
PC1 (50.44%)
H
H9-ANSCs vs H9-PSCs
-Log10 PValue
Log2FC
MYC LIN28A KLF4 POU5F1 NANOG SOX3 NES TUBB3 RARB HOXB3 ASCL1 PAX6 SOX1 RARA NEUROG2 HOXB2 HOXC4 HOXA1 HOXA4 HOXC5
I
Group
H9-PSCs
H9-NSCs
H9-ANSCs
Z-score
POU5F1 NANOG KLF4 MYC LIN28A RARA RARB PAX6 TUBB3 NEUROG2 NES SOX3 ASCL1 CRABP2 SOX1 HOXA1 HOXB3 HOXD1 HOXA4 HOXC4 HOXC5 HOXB2 HOXC9
H9-ANSCs-2 H9-ANSCs-1 H9-ANSCs-3 H9-NSCs-2 H9-NSCs-1 H9-NSCs-3 H9-PSCs1 H9-PSCs-2 H9-PSCs-3
J
axon development
regulation of nervous system development
axonogenesis
regulation of neuron projection development
synapse organization
forebrain development
regulation of neurogenesis
positive regulation of nervous system development
axon guidance
neuron projection guidance
central nervous system neuron differentiation
neuron migration
neural precursor cell proliferation
spinal cord development
neuroblast proliferation
K
Wnt signaling pathway
Calcium signaling pathway
Hedgehog signaling pathway
Notch signaling pathway
Axon guidance
Neurotrophin signaling pathway
Cholinergic synapse
Glutamatergic synapse
GnRH signaling pathway
L
Hedgehog signaling pathway
Enrichment Score
Rank in Ordered Dataset
M
H9-PSCs-3 H9-PSCs-1 H9-PSCs-2 H9-NSCs-1 H9-NSCs-2 H9-NSCs-3 H9-ANSCs-2 H9-ANSCs-1 H9-ANSCs-3
N
H9-NSCs vs H9-PSCs
H9-ANSCs vs H9-PSCs
1798 6808 2270

Fig. S2. Generation and molecular characterization of ESC (H9) and iPSC (Z1) derived ANSCs.

(A), Schematic diagram illustrating the generation of advanced neural stem cells (H9-ANSCs) from pluripotent stem cell line H9 (H9-PSCs) via the intermediate stage of neural stem cells (H9-NSCs). Representative morphology and alkaline phosphatase staining of H9-PSCs, H9-NSCs and H9-ANSCs. Scale bar: 100 μm. **(B)**, Schematic diagram illustrating the generation of advanced neural stem cells (IPS-ANSCs) from induced pluripotent stem cell line Z1 (IPS) via the intermediate stage of neural stem cells (IPS-NSCs). Representative morphology and alkaline phosphatase staining of IPSC, IPS-NSCs and IPS-ANSCs. Scale bar: 100 μm. **(C)**, Immunofluorescence staining for pluripotency markers and lineage-specific markers in H9-PSCs, H9-NSCs and H9-ANSCs. Scale bar: 100 μm. **(D)**, Immunofluorescence staining for pluripotency markers and lineage markers in IPSCs, IPS-NSCs and IPS-ANSCs. Scale bar: 100 μm. **(E)**, RT-qPCR analysis of pluripotency and neuroectodermal genes in H9-PSCs and H9-ANSCs. Data were normalized to *GAPDH*. Error bars represent mean ± SD. (n = 3 biological replicates). *P* values were determined using two-tailed student's t-tests. **(F)**, RT-qPCR analysis of pluripotency and neuroectodermal genes in iPSCs and iPS-ANSCs. Data were normalized to *GAPDH*. Error bars represent mean ± SD. (n = 3 biological replicates). *P* values were determined using two-tailed student's t-tests. **(G)**, Transcriptome-based principal component analysis (PCA) of H9-PSCs, H9-NSCs, H9-ANSCs, ANSCs, PSCs and NSCs. **(H)**, Volcano plot showing differentially expressed genes (DEGs) between H9-ANSCs and H9-PSCs ($|\log_2 FC| > 1$, $P < 0.05$). **(I)**, Heatmap illustrating the expression levels of pluripotency genes, neuroectodermal genes, genes associated with retinoic acid (RA) signaling, and downstream target genes of RA signaling pathway in H9-ANSCs, H9-NSCs and H9-PSCs. **(J)**, Kyoto encyclopedia of genes and genomes (KEGG) enrichment analysis of DEGs between H9-ANSCs and H9-PSCs. **(K)**, Gene ontology (GO) enrichment analysis of DEGs between H9-ANSCs and H9-PSCs. **(L)**, Gene set enrichment analysis (GSEA) plot showing significant enrichment of the hedgehog signaling pathway in DEGs between H9-ANSCs and H9-PSCs. **(M)**, Hierarchical clustering of transcriptomes profiles from H9-PSCs, H9-NSCs, and H9-ANSCs (distance metric: 1- Spearman correlation coefficient). **(N)**, Venn diagram showing the overlap of DEGs between H9-NSCs vs. H9-PSCs and H9-ANSCs vs. H9-PSCs.

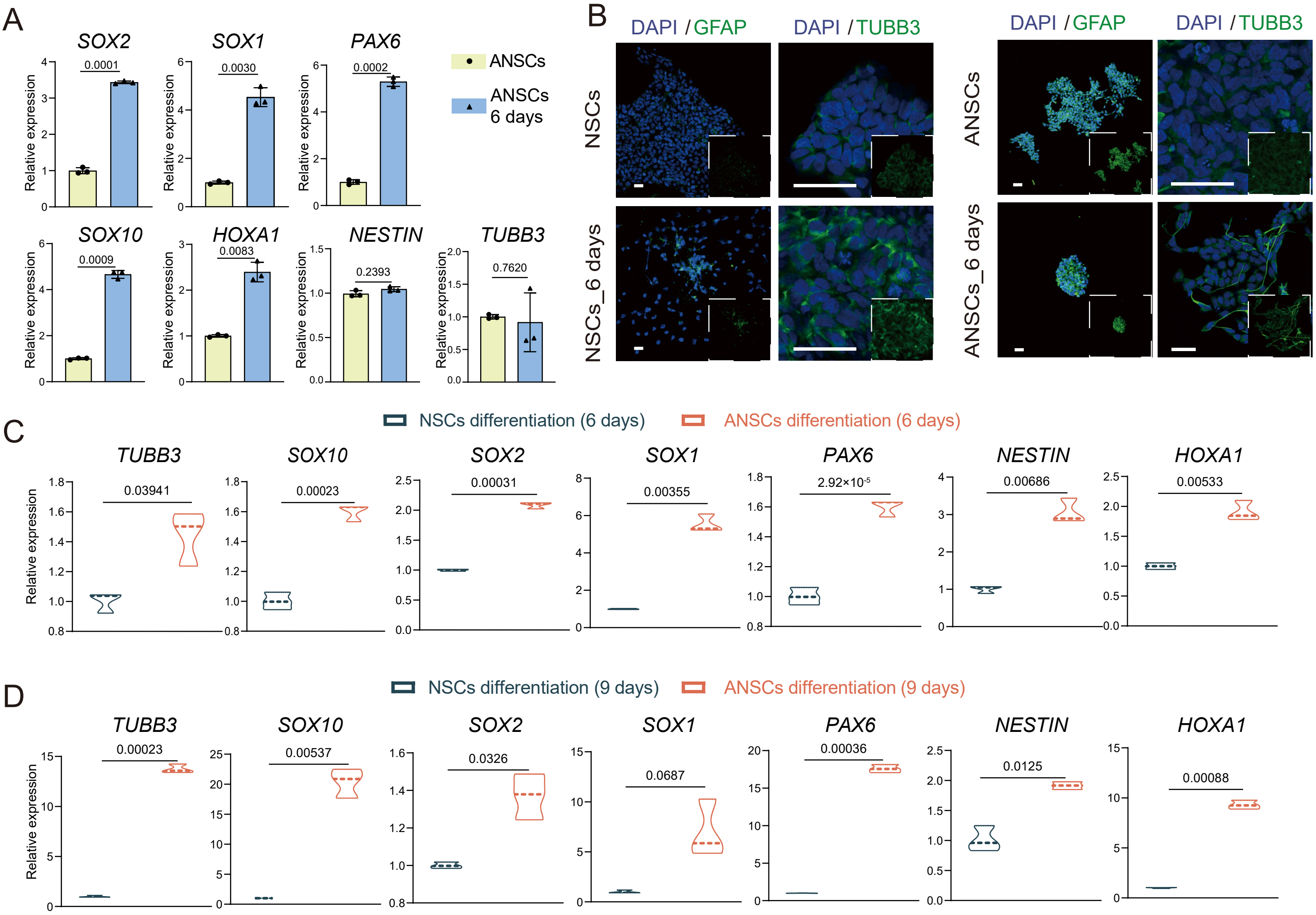
A
SOX2
SOX1
PAX6
SOX10
HOXA1
NESTIN
TUBB3
Relative expression
0.0001
0.0030
0.0002
0.0009
0.0083
0.2393
0.7620
ANSCs
ANSCs 6 days
B
DAPI / GFAP
DAPI / TUBB3
NSCs
NSCs_6 days
ANSCs
ANSCs_6 days
C
NSCs differentiation (6 days)
ANSCs differentiation (6 days)
TUBB3
SOX10
SOX2
SOX1
PAX6
NESTIN
HOXA1
Relative expression
0.03941
0.00023
0.00031
0.00355
2.92×10-5
0.00686
0.00533
D
NSCs differentiation (9 days)
ANSCs differentiation (9 days)
TUBB3
SOX10
SOX2
SOX1
PAX6
NESTIN
HOXA1
Relative expression
0.00023
0.00537
0.0326
0.0687
0.00036
0.0125
0.00088

Fig. S3. ANSCs are biased toward neural lineage differentiation.

(A), RT-qPCR analysis of *SOX2, SOX1, SOX10, HOXA1, PAX6, TUBB3*, and *NESTIN* expression in ANSCs at days 0 and 6 of differentiation. Data were normalized to *GAPDH*. Error bars represent mean ± SD. (n = 3 biological replicates). *P* values were determined using two-tailed student's t-tests. **(B),** Immunofluorescence staining of neuroectodermal markers in NSCs and ANSCs after 6 days of spontaneous differentiation. Scale bar: 50 μm. **(C),** RT-qPCR analysis of *SOX2, SOX1, SOX10, HOXA1, PAX6, TUBB3*, and *NESTIN* expression in NSCs and ANSCs after 6 days of differentiation. Data were normalized to *GAPDH*. Error bars represent mean ± SD. (n = 3 biological replicates). *P* values were determined using two-tailed student's t-tests. **(D),** RT-qPCR analysis of *SOX2, SOX1, SOX10, HOXA1, PAX6, TUBB3*, and *NESTIN* expression in NSCs and ANSCs after 9 days of differentiation. Data were normalized to *GAPDH*. Error bars represent mean ± SD. (n = 3 biological replicates). *P* values were determined using two-tailed student's t-tests.

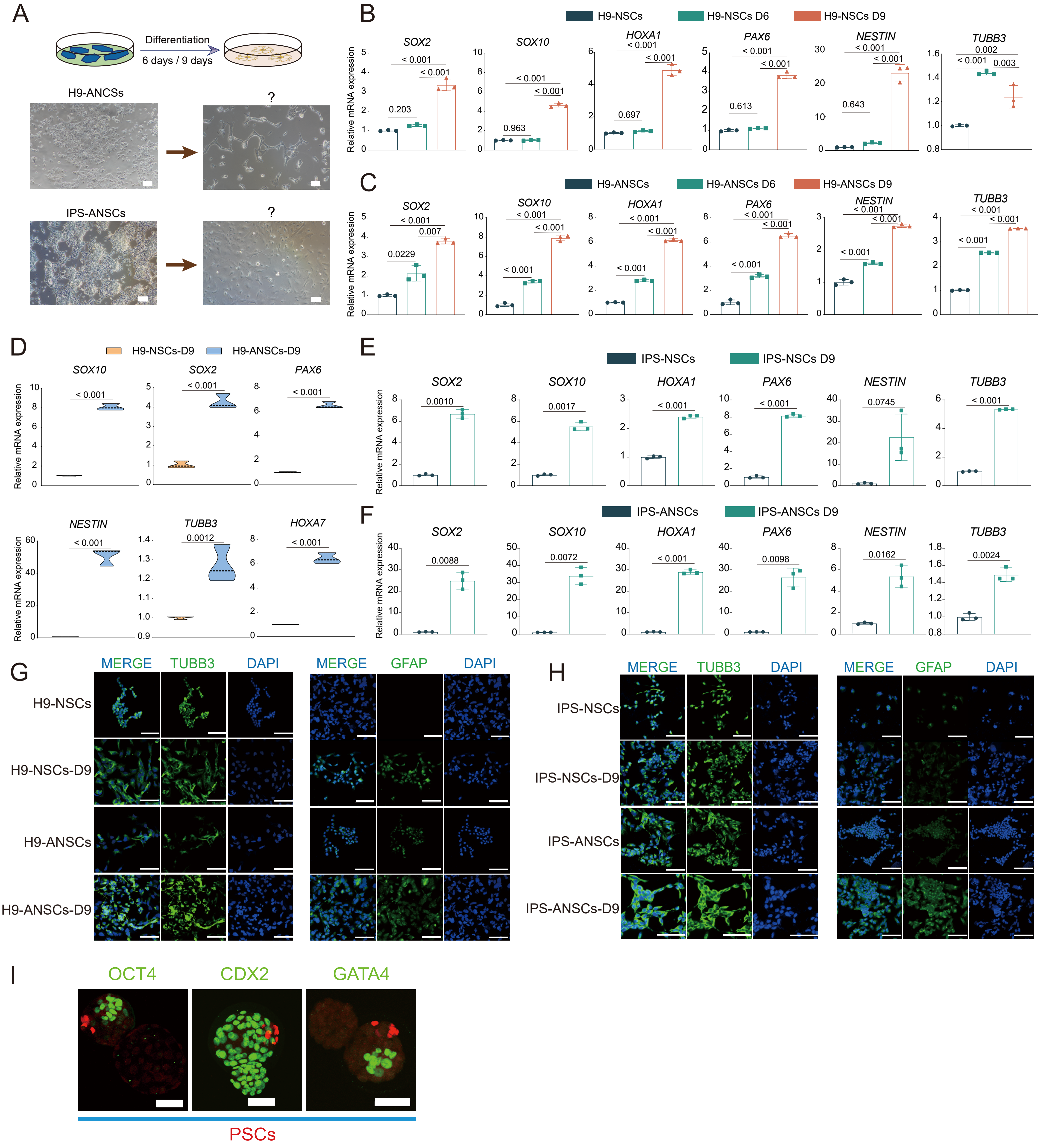
A
Differentiation
6 days / 9 days
H9-ANCSs
?
IPS-ANSCs
?
B
H9-NSCs
H9-NSCs D6
H9-NSCs D9
Relative mRNA expression
SOX2
SOX10
HOXA1
PAX6
NESTIN
TUBB3
0.203
< 0.001
0.963
0.697
0.613
0.643
0.002
0.003
C
H9-ANSCs
H9-ANSCs D6
H9-ANSCs D9
0.0229
0.007
D
H9-NSCs-D9
H9-ANSCs-D9
0.0012
HOXA7
E
IPS-NSCs
IPS-NSCs D9
0.0010
0.0017
0.0745
F
IPS-ANSCs
IPS-ANSCs D9
0.0088
0.0072
0.0098
0.0162
0.0024
G
MERGE
TUBB3
DAPI
GFAP
H9-NSCs
H9-NSCs-D9
H9-ANSCs
H9-ANSCs-D9
H
IPS-NSCs
IPS-NSCs-D9
IPS-ANSCs
IPS-ANSCs-D9
I
OCT4
CDX2
GATA4
PSCs

Fig. S4. Loss of pluripotency and neuroectodermal lineage differentiation during spontaneous differentiation of ESC (H9) and iPSC (Z1) derived ANSCs.

(A), Schematic and morphology on day 9 of H9-ANSCs and IPS-ANSCs during spontaneous differentiation in M10 medium. Scale bar: 100 µm. **(B),** RT-qPCR analysis of *SOX2*, *SOX10*, *HOXA1*, *PAX6*, *NESTIN*, and *TUBB3* expression in H9-NSCs before (Day 0) and after spontaneous differentiation (Day6 and Day 9). Data were normalized to *GAPDH*. Error bars represent mean ± SD. (n = 3 biological replicates). *P* values were determined using two-tailed student's t-tests. **(C),** RT-qPCR analysis of *SOX2*, *SOX10*, *HOXA1*, *PAX6*, *NESTIN*, and *TUBB3* expression in H9-ANSCs before (Day 0) and after spontaneous differentiation (Day6 and Day 9). Data were normalized to *GAPDH*. Error bars represent mean ± SD. (n = 3 biological replicates). *P* values were determined using two-tailed student's t-tests. **(D)**, RT-qPCR analysis of *SOX10*, *SOX2*, *PAX6*, *NESTIN*, *TUBB3*, and *HOXA1* expression in H9-NSCs and H9-ANSCs after 9 days of differentiation. Data were normalized to *GAPDH*. Error bars represent mean ± SD. (n = 3 biological replicates). *P* values were determined using two-tailed student's t-tests. **(E)**, RT-qPCR analysis of *SOX10*, *SOX2*, *PAX6*, *NESTIN*, *TUBB3*, and *HOXA1* expression in IPS-NSCs before (Day 0) and after spontaneous differentiation (Day 9). Data were normalized to *GAPDH*. Error bars represent mean ± SD. (n = 3 biological replicates). *P* values were determined using two-tailed student's t-tests. **(F)**, RT-qPCR analysis of *SOX10*, *SOX2*, *PAX6*, *NESTIN*, *TUBB3*, and *HOXA1* expression in IPS-ANSCs before (Day 0) and after spontaneous differentiation (Day 9). Data were normalized to *GAPDH*. Error bars represent mean ± SD. (n = 3 biological replicates). *P* values were determined using two-tailed student's t-tests. **(G)**, Immunofluorescence staining of TUBB3 and GFAP in H9-NSCs, H9-NSCs after 9 days of spontaneous differentiation, H9-ANSCs, and H9-ANSCs after 9 days of spontaneous differentiation. Scale bar: 100 µm. **(H)**, Immunofluorescence staining of TUBB3 and GFAP in IPS-NSCs, IPS-NSCs after 9 days of spontaneous differentiation, IPS-ANSCs, and IPS-ANSCs after 9 days of spontaneous differentiation. Scale bar: 100 µm. **(I),** Immunofluorescence staining of OCT4, CDX2, and GATA4 in mouse embryos injected with PSCs (W24). Scale bar: 50 µm

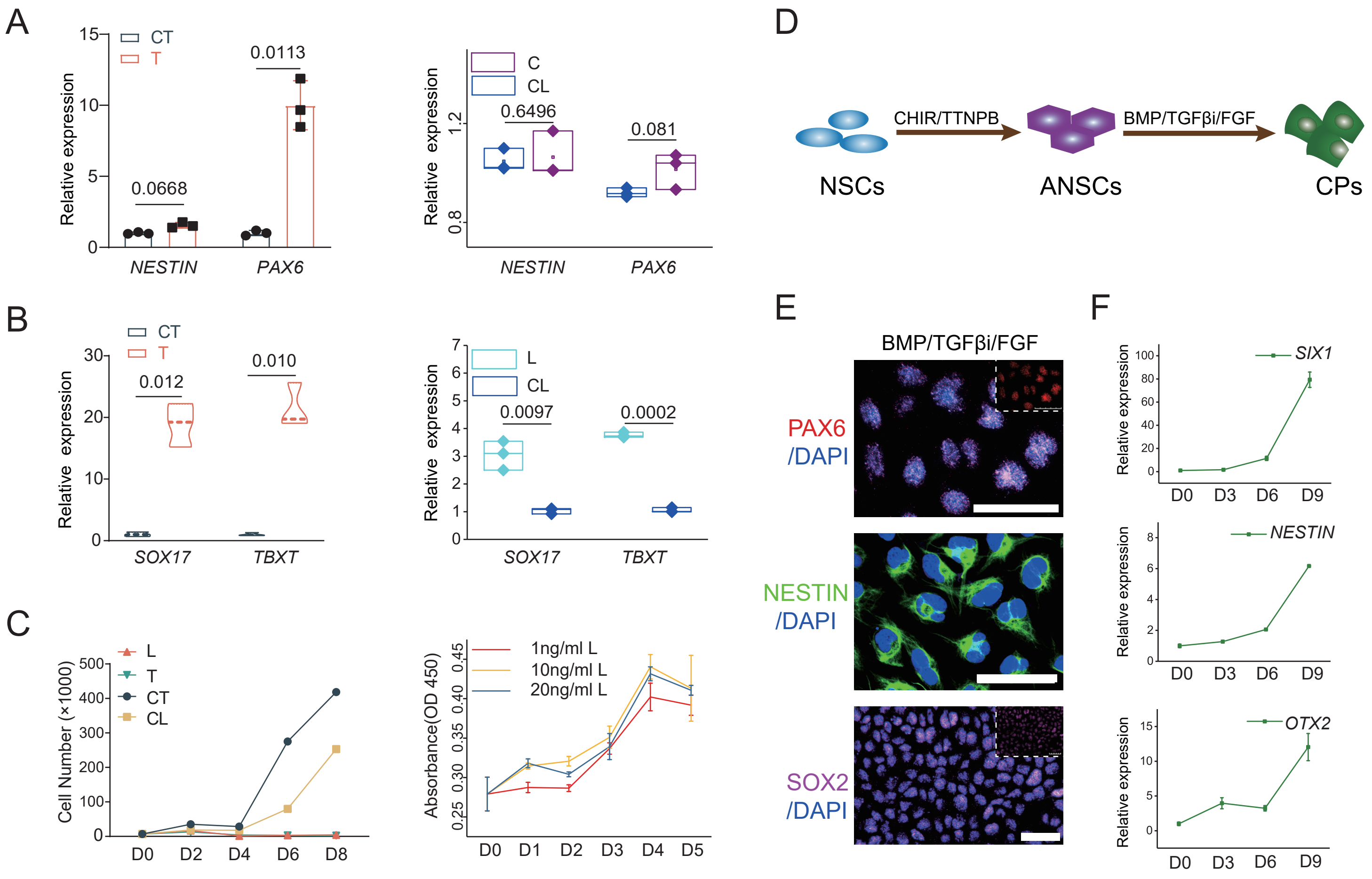
A
CT
T
Relative expression
0.0668
0.0113
NESTIN
PAX6
C
CL
0.6496
0.081
B
SOX17
TBXT
0.012
0.010
L
0.0097
0.0002
C
Cell Number (×1000)
D0
D2
D4
D6
D8
Absorbance(OD 450)
1ng/ml L
10ng/ml L
20ng/ml L
D1
D3
D5
D
CHIR/TTNPB
BMP/TGFβi/FGF
NSCs
ANSCs
CPs
E
PAX6
/DAPI
NESTIN
/DAPI
SOX2
/DAPI
F
SIX1
OTX2
D9

Fig. S5. TTNPB alone is insufficient to support NSC maintenance.

(A), RT-qPCR analysis of the *PAX6* and *NESTIN* expression in ANSCs treated with CHIR99021 plus TTNPB and treated with TTNPB alone (left), and analysis of the *PAX6* and *NESTIN* expression in NSCs treated with CHIR99021 plus LIF and treated with CHIR99021 alone (right). CT: CHIR99021 plus TTNPB; T: TTNPB; C: CHIR99021; CL: CHIR99021 plus leukemia inhibitory factor. Data were normalized to *GAPDH*. Error bars represent mean ± SD. (n = 3 biological replicates). *P* values were determined using two-tailed student's t-tests. **(B),** RT-qPCR analysis of the *SOX17* and *TBXT* expression in ANSCs treated with CHIR99021 plus TTNPB and treated with TTNPB alone (left), and analysis of the *SOX17* and *TBXT* genes in NSCs treated with CHIR99021 plus LIF and treated with CHIR99021 alone (right). CT: CHIR99021 plus TTNPB; T: TTNPB; L: LIF, leukemia inhibitory factor, CL: CHIR99021 plus leukemia inhibitory factor. Data were normalized to *GAPDH*. Error bars represent mean ± SD. (n = 3 biological replicates). *P* values were determined using twotailed student's t-tests. **(C),** Left: Growth curves of PSCs in four culture conditions; Right: Absorbance of NSCs treated with LIF in different concentrations. T: TTNPB; C: CHIR99021; LIF or L: leukemia inhibitory factor; CT: CHIR99021 plus TTNPB; CL: CHIR99021 plus leukemia inhibitory factor. Mean ± SD (n = 2 biological replicates). **(D),** Schematic diagram illustrating the differentiation of NSCs into cranial placode (CP) lineage. **(E),** Immunofluorescence staining of CP markers (PAX6, NESTIN, and SOX2). Scale bar: 50 µm. **(F),** Relative expression of *SIX1, NESTIN,* and *OTX2* in CP cells at different time points.

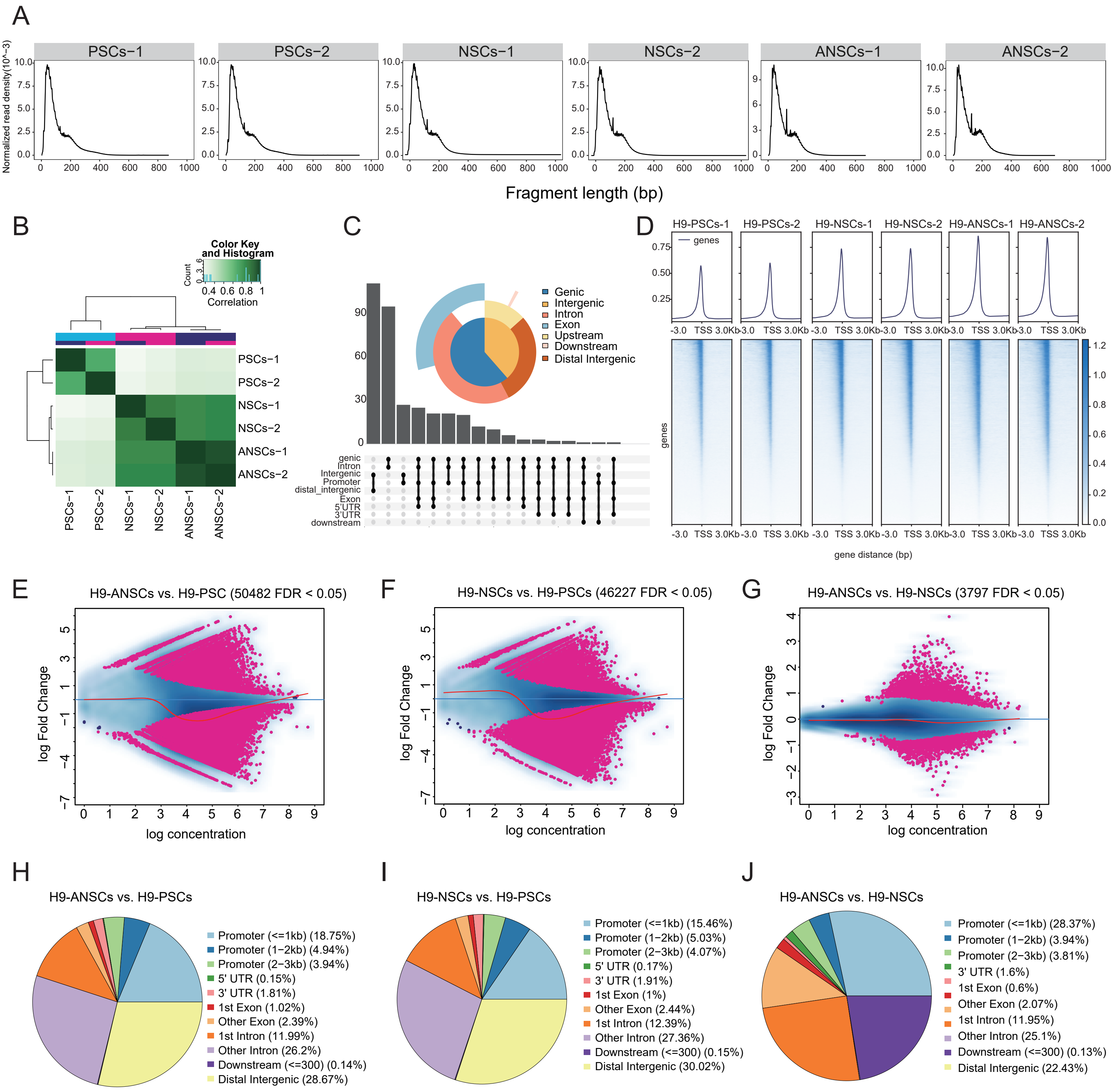
A
PSCs−1
PSCs−2
NSCs−1
NSCs−2
ANSCs−1
ANSCs−2
Normalized read density(10^-3)
Fragment length (bp)
B
Color Key and Histogram
Count
Correlation
PSCs−1
PSCs−2
NSCs−1
NSCs−2
ANSCs−1
ANSCs−2
C
Genic
Intergenic
Intron
Exon
Upstream
Downstream
Distal Intergenic
genic
Intron
Intergenic
Promoter
distal_intergenic
Exon
5'UTR
3'UTR
downstream
D
H9-PSCs-1
H9-PSCs-2
H9-NSCs-1
H9-NSCs-2
H9-ANSCs-1
H9-ANSCs-2
genes
-3.0 TSS 3.0Kb
gene distance (bp)
E
H9-ANSCs vs. H9-PSC (50482 FDR < 0.05)
log Fold Change
log concentration
F
H9-NSCs vs. H9-PSCs (46227 FDR < 0.05)
log Fold Change
log concentration
G
H9-ANSCs vs. H9-NSCs (3797 FDR < 0.05)
log Fold Change
log concentration
H
H9-ANSCs vs. H9-PSCs
Promoter (<=1kb) (18.75%)
Promoter (1−2kb) (4.94%)
Promoter (2−3kb) (3.94%)
5' UTR (0.15%)
3' UTR (1.81%)
1st Exon (1.02%)
Other Exon (2.39%)
1st Intron (11.99%)
Other Intron (26.2%)
Downstream (<=300) (0.14%)
Distal Intergenic (28.67%)
I
H9-NSCs vs. H9-PSCs
Promoter (<=1kb) (15.46%)
Promoter (1−2kb) (5.03%)
Promoter (2−3kb) (4.07%)
5' UTR (0.17%)
3' UTR (1.91%)
1st Exon (1%)
Other Exon (2.44%)
1st Intron (12.39%)
Other Intron (27.36%)
Downstream (<=300) (0.15%)
Distal Intergenic (30.02%)
J
H9-ANSCs vs. H9-NSCs
Promoter (<=1kb) (28.37%)
Promoter (1−2kb) (3.94%)
Promoter (2−3kb) (3.81%)
3' UTR (1.6%)
1st Exon (0.6%)
Other Exon (2.07%)
1st Intron (11.95%)
Other Intron (25.1%)
Downstream (<=300) (0.13%)
Distal Intergenic (22.43%)

Fig. S6. High-quality ATAC-seq profiling reveals epigenetic remodeling underlies the transition from PSCs to ANSCs.

(A), Distribution of fragment length in PSCs, NSCs, and ANSCs. **(B),** Heatmap showing the correlation matrix among PSCs, NSCs, and ANSCs. **(C),** Genomic distribution of differential ATAC-seq peaks (ANSCs vs. NSCs). **(D)**, ATAC-seq analysis of H9-PSCs, H9-NSCs, and H9-ANSCs. ATAC-seq signals at Refseq genes as normalized CPM (counts per million). Heatmaps showing the landscapes of peaks around the transcription start sites (TSSs). **(E-G)**, MA plots of differential chromatin accessibility in **(E)** H9-ANSC vs. H9-PSCs, **(F)** H9-NSCs vs. H9-PSCs, and **(G)** H9-ANSCs vs. H9-NSCs. **(H-J)**, Genomic distribution of differential ATAC-seq peaks of **(H)** H9-ANSCs vs. H9-PSCs, **(I)** H9-NSCs vs. H9-PSCs, and **(J)** H9-ANSCs vs. H9-NSCs.

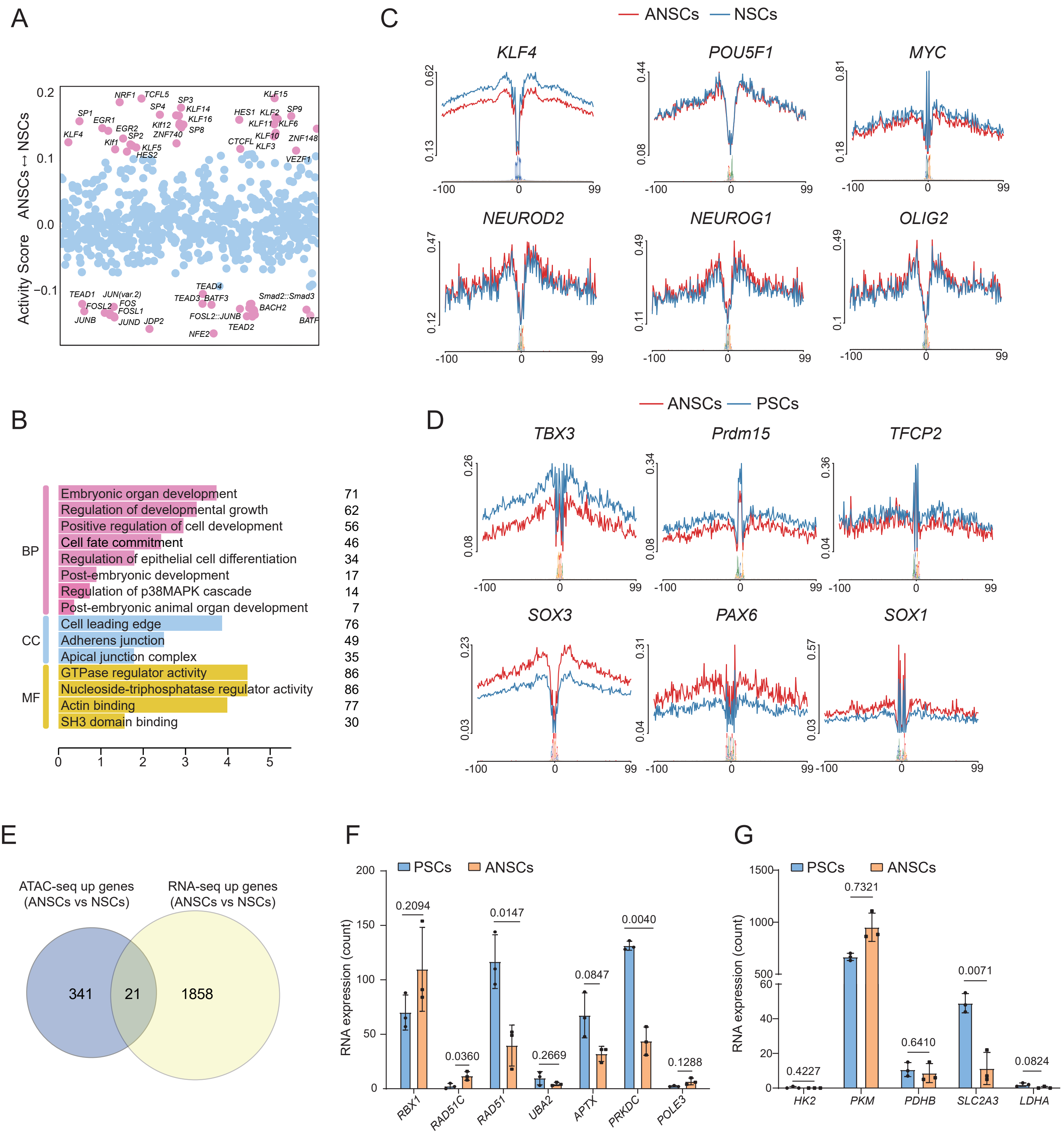

A
Activity Score ANSCs ↔ NSCs
0.2
0.1
0.0
−0.1
NRF1 TCFL5 SP3 SP4 KLF14 KLF16 SP8 SP1 EGR1 EGR2 Klf12 ZNF740 KLF4 Klf1 SP2 KLF5 HES2 KLF15 HES1 KLF2 SP9 KLF11 KLF6 KLF10 ZNF148 CTCFL KLF3 VEZF1
TEAD1 JUN(var.2) FOSL2 FOS FOSL1 JUNB JUND JDP2 TEAD4 TEAD3 BATF3 FOSL2::JUNB NFE2 Smad2::Smad3 BACH2 TEAD2 BATF
B
BP
Embryonic organ development 71
Regulation of developmental growth 62
Positive regulation of cell development 56
Cell fate commitment 46
Regulation of epithelial cell differentiation 34
Post-embryonic development 17
Regulation of p38MAPK cascade 14
Post-embryonic animal organ development 7
CC
Cell leading edge 76
Adherens junction 49
Apical junction complex 35
MF
GTPase regulator activity 86
Nucleoside-triphosphatase regulator activity 86
Actin binding 77
SH3 domain binding 30
0 1 2 3 4 5
C
ANSCs NSCs
KLF4 POU5F1 MYC NEUROD2 NEUROG1 OLIG2
-100 0 99
D
ANSCs PSCs
TBX3 Prdm15 TFCP2 SOX3 PAX6 SOX1
-100 0 99
E
ATAC-seq up genes (ANSCs vs NSCs)
RNA-seq up genes (ANSCs vs NSCs)
341 21 1858
F
PSCs ANSCs
RNA expression (count)
0.2094 0.0360 0.0147 0.2669 0.0847 0.0040 0.1288
RBX1 RAD51C RAD51 UBA2 APTX PRKDC POLE3
G
PSCs ANSCs
RNA expression (count)
0.4227 0.7321 0.6410 0.0071 0.0824
HK2 PKM PDHB SLC2A3 LDHA

Fig. S7. Conversion of PSCs to ANSCs induces altered chromatin accessibility characteristics without DNA damage or glycolytic dysfunction

(A), Scatter plot of chromatin accessibility activity scores for ANSCs vs. NSCs (each point represents one gene). **(B),** GO enrichment analysis of genes with increased chromatin accessibility (ANSCs vs. NSCs). BP: biological process; CC: cellular component; MF: molecular function. **(C, D),** Chromatin footprints of pluripotent and neuroectodermal transcription factors in **(C)** ANSCs vs. NSCs and **(D)** ANSCs vs. PSCs. **(E),** Venn diagram showing overlap between upregulated genes (ANSCs vs. NSCs) from ATAC-seq and RNA-seq data. **(F),** Gene expression of the DNA damage markers in PSCs and ANSCs. Error bars represent mean ± SD. (n = 3 biological replicates). *P* values were determined using two-tailed student's t-tests. **(G),** Gene expression of the glycolysis-related markers in PSCs and ANSCs. Error bars represent mean ± SD. (n = 3 biological replicates). *P* values were determined using two-tailed student's t-tests.

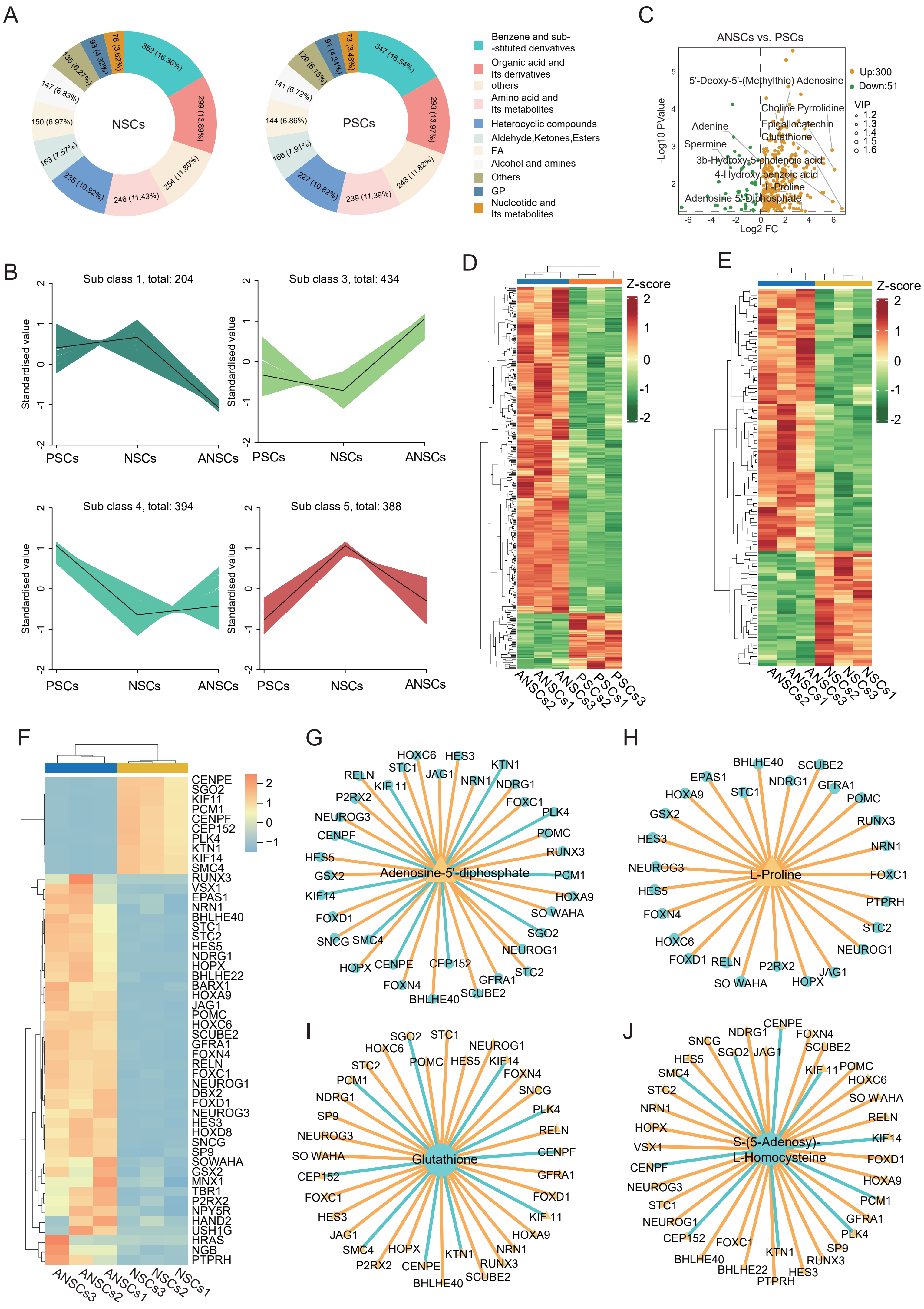

A
NSCs
PSCs
352 (16.36%)
299 (13.89%)
254 (11.80%)
246 (11.43%)
235 (10.92%)
163 (7.57%)
150 (6.97%)
147 (6.83%)
135 (6.27%)
93 (4.32%)
78 (3.62%)
347 (16.54%)
293 (13.97%)
248 (11.82%)
239 (11.39%)
227 (10.82%)
166 (7.91%)
144 (6.86%)
141 (6.72%)
129 (6.15%)
91 (4.34%)
73 (3.48%)
Benzene and sub- -stituted derivatives
Organic acid and Its derivatives
others
Amino acid and Its metabolites
Heterocyclic compounds
Aldehyde,Ketones,Esters
FA
Alcohol and amines
Others
GP
Nucleotide and Its metabolites
C
ANSCs vs. PSCs
5'-Deoxy-5'-(Methylthio) Adenosine
Choline Pyrrolidine
Adenine
Epigallocatechin
Glutathione
Spermine
3b-Hydroxy-5-cholenoic acid
4-Hydroxy benzoic acid
L-Proline
Adenosine 5'-Diphosphate
Up:300
Down:51
VIP
1.2
1.3
1.4
1.5
1.6
-Log10 PValue
Log2 FC
B
Sub class 1, total: 204
Sub class 3, total: 434
Sub class 4, total: 394
Sub class 5, total: 388
Standardised value
PSCs
NSCs
ANSCs
D
Z-score
ANSCs2
ANSCs1
ANSCs3
PSCs2
PSCs1
PSCs3
E
Z-score
ANSCs2
ANSCs1
ANSCs3
NSCs2
NSCs3
NSCs1
F
CENPE
SGO2
KIF11
PCM1
CENPF
CEP152
PLK4
KTN1
KIF14
SMC4
RUNX3
VSX1
EPAS1
NRN1
BHLHE40
STC1
STC2
HES5
NDRG1
HOPX
BHLHE22
BARX1
HOXA9
JAG1
POMC
HOXC6
SCUBE2
GFRA1
FOXN4
RELN
FOXC1
NEUROG1
DBX2
FOXD1
NEUROG3
HES3
HOXD8
SNCG
SP9
SOWAHA
GSX2
MNX1
TBR1
P2RX2
NPY5R
HAND2
USH1G
HRAS
NGB
PTPRH
ANSCs3
ANSCs2
ANSCs1
NSCs3
NSCs2
NSCs1
G
Adenosine-5'-diphosphate
H
L-Proline
I
Glutathione
J
S-(5-Adenosy)-
L-Homocysteine

Fig. S8. ANSCs display a unique metabolomic signature.

(A), Metabolite class distribution identified in NSCs and PSCs. Pie charts illustrating the proportion of per class. **(B),** *K*-means clustering of differentially abundant metabolites across PSCs, NSCs, and ANSCs. **(C),** Volcano plot showing differential metabolite abundance (ANSCs vs. PSCs; $|\log2FC| > 1$, $P < 0.05$). **(D),** Heatmap showing the difference of metabolites between ANSCs and PSCs. **(E),** Heatmap showing the difference of metabolites between ANSCs and NSCs. **(F),** Heatmap showing gene expression profiles of ANSCs and NSCs. **(G-J),** Network of Spearman correlations between ADP **(G)**, L-proline **(H)**, glutathione **(I)**, SAH **(J)**, and neural-associated genes. Yellow lines: positive correlations; Blue lines: negative correlations. ADP: adenosine 5′-diphosphate, SAH: S-(5′-adenosyl)-L-homocysteine.

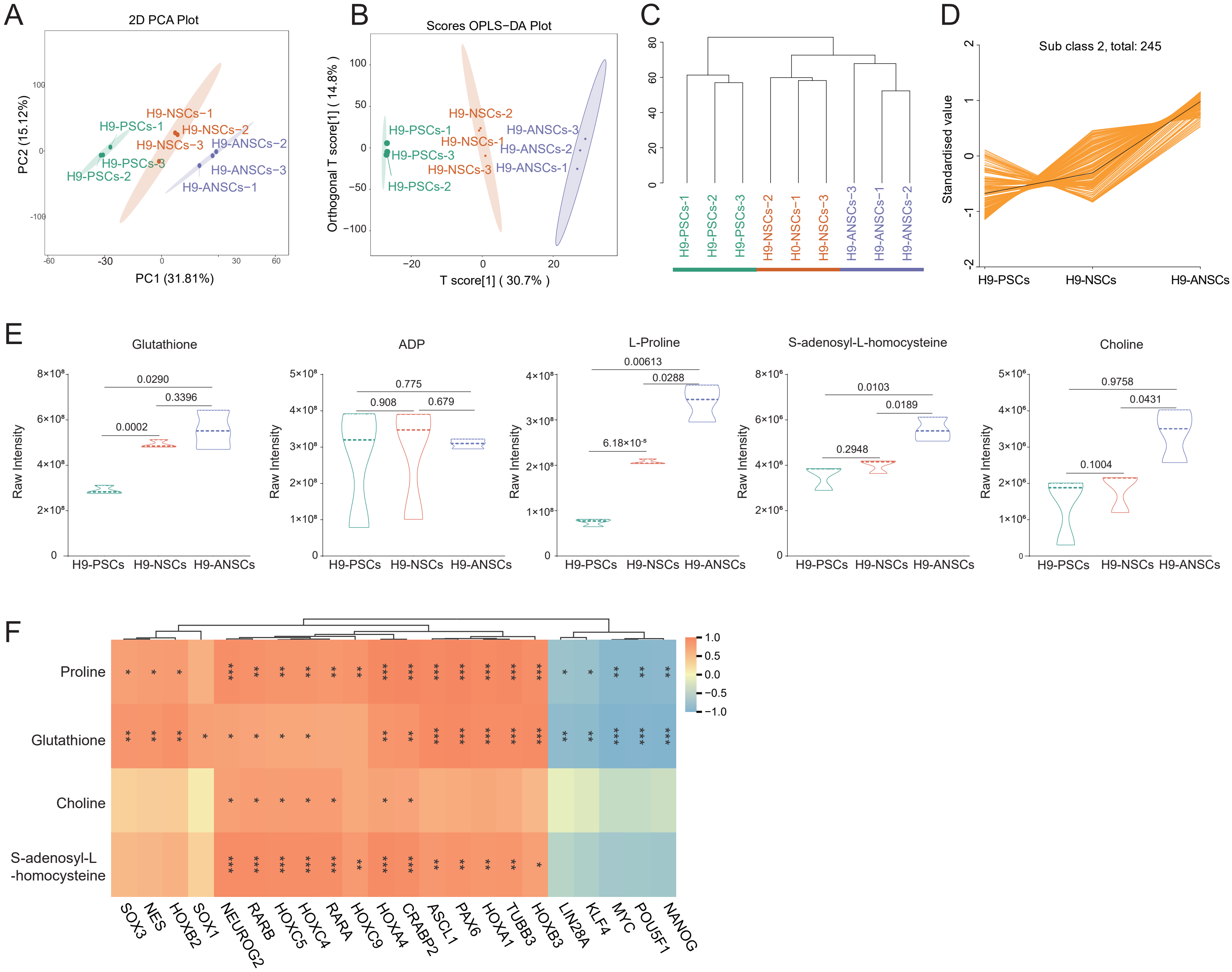
A
2D PCA Plot
PC2 (15.12%)
PC1 (31.81%)
H9-PSCs-1
H9-PSCs-2
H9-PSCs-3
H9-NSCs−1
H9-NSCs−2
H9-NSCs−3
H9-ANSCs−1
H9-ANSCs−2
H9-ANSCs−3
B
Scores OPLS−DA Plot
Orthogonal T score[1] (14.8%)
T score[1] (30.7%)
C
H9-PSCs-1
H9-PSCs-2
H9-PSCs-3
H9-NSCs−2
H0-NSCs−1
H9-NSCs−3
H9-ANSCs−3
H9-ANSCs−1
H9-ANSCs−2
D
Sub class 2, total: 245
Standardised value
H9-PSCs
H9-NSCs
H9-ANSCs
E
Glutathione
ADP
L-Proline
S-adenosyl-L-homocysteine
Choline
Raw Intensity
0.0290
0.3396
0.0002
0.775
0.908
0.679
0.00613
0.0288
6.18×10⁻⁶
0.0103
0.0189
0.2948
0.9758
0.0431
0.1004
F
Proline
Glutathione
Choline
S-adenosyl-L
-homocysteine
SOX3
NES
HOXB2
SOX1
NEUROG2
RARB
HOXC5
HOXC4
RARA
HOXC9
HOXA4
CRABP2
ASCL1
PAX6
HOXA1
TUBB3
HOXB3
LIN28A
KLF4
MYC
POU5F1
NANOG

Fig S9. Metabolic reprogramming during the transition from H9-PSCs to H9-NSCs and H9-ANSCs.

(A), PCA plot of metabolites from H9-PSCs, H9-NSCs, and H9-ANSCs. **(B),** Orthogonal partial least squares discriminant analysis (OPLS-DA) of metabolites from H9-PSCs, H9-NSCs, and H9-ANSCs. **(C),** Hierarchical clustering of metabolites profiles from H9-PSCs, H9-NSCs, and H9-ANSCs. **(D),** *K*-means clustering of differentially abundant metabolites across H9-PSCs, H9-NSCs, and H9-ANSCs. **(E),** Violin plots showing the relative abundance (raw peak area) of five key metabolites in H9-PSCs, H9-NSCs, and H9-ANSCs. *P* values were determined using two-tailed student's t-tests. **(F),** Heatmap showing pearson correlations between metabolite abundance and the expression of neuroectodermal and pluripotent genes (Z-scores). Orange shows positive correlations. Blue shows negative correlations. $*P < 0.05$, $**P < 0.01$, $***P < 0.001$.

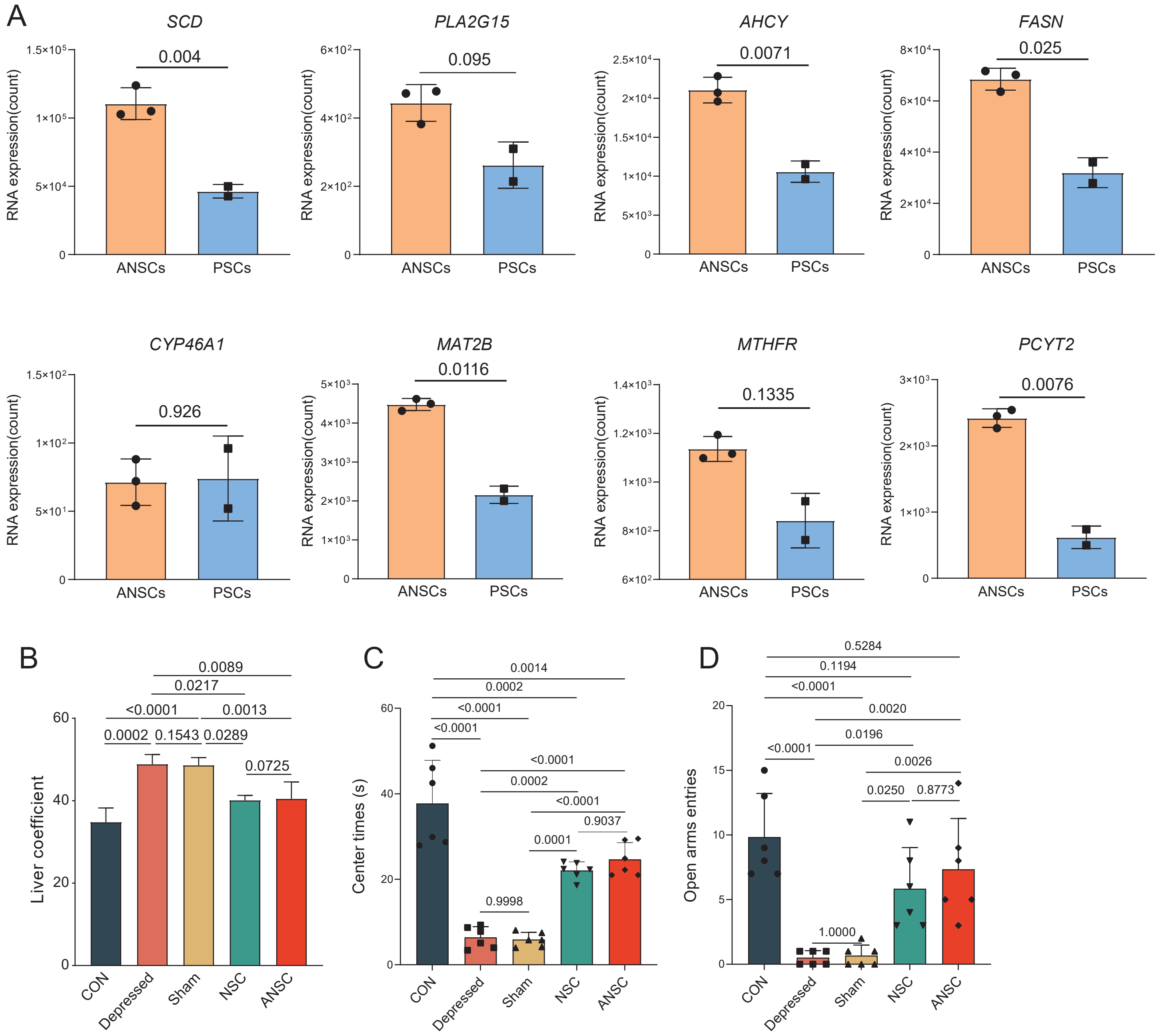

A
SCD
0.004
PLA2G15
0.095
AHCY
0.0071
FASN
0.025
CYP46A1
0.926
MAT2B
0.0116
MTHFR
0.1335
PCYT2
0.0076
RNA expression(count)
ANSCs
PSCs
B
Liver coefficient
0.0089
0.0217
<0.0001
0.0013
0.0002
0.1543
0.0289
0.0725
CON
Depressed
Sham
NSC
ANSC
C
Center times (s)
0.0014
0.0002
<0.0001
<0.0001
<0.0001
0.0002
<0.0001
0.9037
0.0001
0.9998
D
Open arms entries
0.5284
0.1194
<0.0001
0.0020
0.0196
<0.0001
0.0026
0.0250
0.8773
1.0000

Fig. S10. Genes involved in PE-PC metabolism are significantly upregulated in ANSCs and evaluation of ANSC-transplanted depression model rats

(A), Expression of genes involved in PE to PC to choline metabolic process. PE: phosphatidylethanolamine; PC: phosphatidylcholine. *P* values were determined using two-tailed student's t-tests. **(B),** Liver coefficient of rats from each group at the experimental endpoint (day 28, n = 6). *P* values were determined using Welch's t-tests. **(C),** Total time spent in the center zone during the OFT. *P* values were determined using Welch's t-tests. **(D),** Total entries into the open arms of the EPM in each group. *P* values were determined using Welch's t-tests.

Supplementary Table S1 : Primer Sequences Used for RT-qPCR

Gene ID	Symbol	Primer Direction	Sequence (5′–3′)
2597	GAPDH	Forward	CTCTGCTCCTCCTGTTCGAC
		Reverse	TTAAAAGCAGCCCTGGTGAC
5460	OCT4	Forward	ATGCATTCAAACTGAGGTGCCTGC
		Reverse	CCCTTTGTGTTCCCAATTCCTTCC
6657	SOX2	Forward	GCTGCAAAAGAGAACACCAATCCC
		Reverse	AAACTTCCTGCAAAGCTCCTACCG
77923	NANOG	Forward	GGTAGAAATTGGGGTTTAGAAAT
		Reverse	TACAAAAAACAAACAACTTCCC
10763	NESTIN	Forward	GCACCTCAAGATGTCCCTCAG
		Reverse	CTGGGAGCAAAGATCCAAGAC
5080	PAX6	Forward	GTACTGAATGACTCAACTGCTCGG
		Reverse	CTTTAGAAGGAAGCGACACTCTGC
6656	SOX1	Forward	CAACCAGGACCGGGTCAAACG
		Reverse	GCCTCGGACATGACCTTCCACT
6663	SOX10	Forward	ACCTGGTCTTCCAGCCCTAT
		Reverse	CAGGGGCCAGATGGGTTTAG
3198	HOXA1	Forward	CTCAAGTTGTGGTCCAAGCTAT
		Reverse	TGGGTCTGCTTCCTGATTTAAC
3201	HOXA4	Forward	TCCACTTCAATCGATACCTGAC
		Reverse	TCTTTCTTCCACTTCATCCTCC
3204	HOXA7	Forward	ACTACCTATTTTGTGCTGGCTGGC
		Reverse	GAGAAGGAGGGATTGATTCTAGGG
3211	HOXB1	Forward	GGTCAAGATTTGGTTCCAGAACCG
		Reverse	ATTGGTGGCTAGGTTCAGTTCAGG
10381	TUBB3	Forward	GCCTGACAATTTCATCTTTGGTC
		Reverse	CAGTCGCAGTTTTCACACTCCTT
146713	NeuN	Forward	CCGAGTGATGACCAACAAGAAG
		Reverse	CGCAGCCCGAAATGTATTATAC

Gene ID	Symbol	Primer Direction	Sequence (5′–3′)
5914	RARα	Forward	AGCACCAGCTTCCAGTTAGTGG
		Reverse	CAAAGCAAGGCTTGTAGATGCGG
5915	RARβ	Forward	TGAGTGAGGTTCAGTGTCGC
		Reverse	TTCCCTTTTTGCAGCATGGC
5916	RARγ	Forward	CATCACCAAGGTCAGCAAAGCC
		Reverse	GCTCACTGAACTTGTCCCACAG
1062	CENPE	Forward	GCTGGTGACCTCTTCTTCCC
		Reverse	ACTTCTGCATGCTTAACTAAATTCT
151246	SGO2	Forward	CCGGAGAACCCAAAAATCAGG
		Reverse	TGAAGGGAGAGAAAACGGGC
3832	KIF11	Forward	TGTCCAAAGAAGAGCTTAGGGT
		Reverse	GTAAGGGGCATGTCTGCTGT
5108	PCM1	Forward	TTGAAGTGTGGAGCGGGAAA
		Reverse	GTTGGGCACCCCAATCCATA
22995	CEP152	Forward	ACTAGAGCCAGGGTAAGGGG
		Reverse	ACTGGAGCTCTGGAGAGGAG
10733	PLK4	Forward	CGGAAGGTGTCAGGGAGAAC
		Reverse	GATCTTCTCCCCGATGCAGG
3895	KTN1	Forward	ATGAGGCAGCACAGCAAGAT
		Reverse	CTCATTTGCCAGGGCCTGTA
9928	KIF14	Forward	ACGAACTGGAGGATTGGCTG
		Reverse	GTTGTCCTTTTTGCTGCCCC
10051	SMC4	Forward	GAACAGCATTCCTCCTCCCC
		Reverse	ACTGCCATTTGGCCCGATAA
50674	NEUROG3	Forward	AAAGGACCTGTCTGTCGCTG
		Reverse	GTGTAAAACAGGGGAGGGGC
3205	HOXA9	Forward	GGGCCCTGGGCAACTACTA
		Reverse	GTGGCCTGAGGTTTAGAGCC
100131390	SP9	Forward	CGAGCACACAAGCGAGTAGA

Gene ID	Symbol	Primer Direction	Sequence (5′–3′)
		Reverse	CTCGAGAAGTTTGGGCGAGT
30813	VSX1	Forward	CGAAGACCGGATACAGTGCAA
		Reverse	AAAGCCCGGGTTGAGATCAG
5649	RELN	Forward	ATCTGCATCTGCGACGAGAG
		Reverse	CGACCTCCACATGGTCCAAA
390992	HES3	Forward	CCGAAAGATTTCCAAGCCGC
		Reverse	GAGCTGCTCCAGTGACACAT
440097	DBX2	Forward	ACTCTAATTCCAAAGCTCGGAGG
		Reverse	GGCAAGTTTCTTTCGGTCTGTTT
1063	CENPF	Forward	CTCTCCCGTCAACAGCGTTC
		Reverse	GTTGTGCATATTCTTGGCTTGC
3223	HOXC6	Forward	ACAGACCTCAATCGCTCAGGA
		Reverse	AGGGGTAAATCTGGATACTGGC
121643	FOXN4	Forward	ATGAGGGATTCAGCTTGGACA
		Reverse	GACCCGTCACCTGCAAGTC
4762	NEUROG1	Forward	GCTCTCTGACCCCAGTAGC
		Reverse	GCGTTGTGTGGAGCAAGTC

Supplementary Table S2 : Human-specific gene sequences for align the raw RNA-sequencing data of rat hippocampal samples

Symbol	Primer Direction	Sequence (5′–3′)
TBC1D31	Forward	AACCCAGGTTCCAAAATGAACAGGACTCAAGCTGTTTGCCTAGAACC
	Reverse	GGTTCTAGGCAAACAGCTTGAGTCCTGTTCATTTTGGAACCTGGGTT
NOTCH2NLC	Forward	CCAGGACACTGCCAGCATGGTGGCACCTGCCTCAACCTGCCTGGTTCCTACCA
	Reverse	TGGTAGGAACCAGGCAGGTTGAGGCAGGTGCCACCATGCTGGCAGTGTCCTGG
TRIM52	Forward	GGCCATCTGTGTGGTGTGCCGAGAATCCAGGAGCCACAAACAGCACAGCGT
	Reverse	ACGCTGTGCTGTTTGTGGCTCCTGGATTCTCGGCACACCACACAGATGGCC
SRGAP2C	Forward	CAACCAGCAAGAGACAGAGCAGTTTTATTTCACAGTAAGGGAGTGCTATGGCTTT
	Reverse	AAAGCCATAGCACTCCCTTACTGTGAAATAAAACTGCTCTGTCTCTTGCTGGTTG
